# Maternal Exposure to Wood-Smoke-Derived PM_2.5_ Is Associated with Delayed Fetal Neurocranial Intramembranous Ossification in a Rat Model

**DOI:** 10.3390/ijms27135715

**Published:** 2026-06-24

**Authors:** Paulo Salinas, Francisca Villarroel, Luis Astorga, Paula Cerda, Eva Rojas, Aliro Maulén

**Affiliations:** 1 Laboratory of Animal and Experimental Morphology, Institute of Biology, Faculty of Sciences, Pontificia Universidad Católica de Valparaíso, Avenida Universidad 330, Valparaiso 2373223, Chile; 2 Morphohistology Unit, Facultad de Ciencias de la Vida, Universidad de Viña del Mar, Viña del Mar 2520000, Chile

**Keywords:** PM2.5, wood smoke, intramembranous ossification, fetal development, neurocranium, HIF-1α, collagen type I, bone mineral density, developmental toxicology

## Abstract

Maternal exposure to airborne particulate matter smaller than 2.5 μm (${\mathrm{PM}}_{2.5}$) has been associated with adverse fetal outcomes, although its effects on intramembranous ossification remain poorly understood. This study evaluated the impact of gestational and pregestational exposure to wood-smoke-derived ${\mathrm{PM}}_{2.5}$ on fetal neurocranial ossification in Sprague–Dawley rats. Females were allocated to four exposure conditions combining filtered air (FA) and non-filtered air (NFA): FA/FA, FA/NFA, NFA/FA, and NFA/NFA. Fetuses were collected at gestational day 21 and analyzed using fetal morphometry, radiography, micro-computed tomography, whole-mount alizarin red skeletal staining, histology, and immunohistochemistry for HIF-1α, COL-1, BMP-2, FGF-R1, and TGF-β. Continuous exposure (NFA/NFA) was associated with reduced fetal weight, shorter crown–rump length, impaired craniofacial mineralization, widened cranial sutural regions, and reduced mineral density, particularly in the occipital and interparietal bones. Histologically, exposed fetuses exhibited abundant osteoid, reduced osteocyte incorporation, and diffuse osteoblastic distribution, consistent with delayed osteogenic maturation. Immunohistochemistry showed increased HIF-1α immunoreactivity, altered TGF-β regulation, and reduced COL-1 expression in continuously exposed fetuses, whereas BMP-2 and FGF-R1 showed no significant changes. These findings suggest that maternal exposure to wood-smoke-derived PM_2.5_ is associated with delayed fetal neurocranial intramembranous ossification, particularly under continuous exposure. The observed immunohistochemical profile, elevated HIF-1α, reduced COL-I, and altered TGF-β, is consistent with a hypoxia-associated imbalance between extracellular matrix deposition and mineral maturation; however, the underlying mechanistic pathway was not directly functionally tested and should be regarded as a biologically plausible inferential model requiring further experimental validation.

## 1. Introduction

Maternal exposure to atmospheric pollutants during gestation is associated with intrauterine growth restriction, preterm birth, and organogenesis disturbances [1,2]. Fine particulate matter (${\mathrm{PM}}_{2.5}$; ≤2.5 μm) particles or particle-associated components may reach the placental compartment [3], inducing oxidative stress, inflammation, and endocrine disruption that compromise tissue homeostasis [4,5]. Prenatal ${\mathrm{PM}}_{2.5}$ exposure reduces fetal femoral length [1] and increases craniofacial anomaly risk [2], positioning ${\mathrm{PM}}_{2.5}$ as an early-life determinant of musculoskeletal health within the developmental origins of health and disease (DOHaD) framework.

The developing cranial vault is particularly susceptible to hypoxic and oxidative disturbances given its high metabolic demands and tightly coordinated molecular signaling. Intramembranous ossification generates flat skull bones through direct mesenchymal-to-osteoblast differentiation in a highly vascularized microenvironment, lacking cartilaginous intermediates [6,7]. Its molecular network involves RUNX2, OSX, BMPs, TGF-β, FGFs, and type I collagen (COL-I), whose dysregulation compromises neurocranial morphogenesis [6]. COL-I constitutes the primary osteoid matrix component, with TGF-β regulating osteoprogenitor proliferation and differentiation [6]. Osteoblast commitment and mineralization critically depend on oxygen availability; hypoxia stabilizes HIF-1α and induces TWIST, repressing RUNX2 and BMP2 expression and suppressing osteoblast differentiation [8]. Hypoxic stress downregulates COL-I transcription and disrupts TGF-β signaling, compromising matrix deposition and mineralization [8]. This axis provides a biologically plausible framework through which ${\mathrm{PM}}_{2.5}$-associated placental dysfunction may contribute to impaired fetal cranial ossification.

In southern Chile, residential wood smoke constitutes the dominant ${\mathrm{PM}}_{2.5}$ source, with winter concentrations exceeding WHO recommendations and a toxicological profile (polycyclic aromatic hydrocarbons, aldehydes, heavy metals) distinct from urban-traffic-derived ${\mathrm{PM}}_{2.5}$. Gestational exposure induces placental hypoxia, microvascular remodeling, increased HIF-1α, VEGF, and Flt-1, reduced fetal growth [9], and impaired GLUT1 and SVCT2 transporters compromising glucose and vitamin C delivery [10]. Effects on fertility, folliculogenesis, and fetal anthropometry have been documented [11]. In mice, maternal ${\mathrm{PM}}_{2.5}$ inhibits spiral artery remodeling, generating an unfavorable intrauterine environment for mineralization [12]. In humans, gestational ${\mathrm{PM}}_{2.5}$ deteriorates maternal bone strength [13], with Mendelian randomization analyses supporting a causal link between ${\mathrm{PM}}_{2.5}$ and reduced bone mineral density [14].

Despite convergent epidemiological and experimental evidence linking gestational ${\mathrm{PM}}_{2.5}$ exposure to impaired fetal growth and altered skeletal development, most available studies have focused on endochondral skeletal elements, postnatal growth, or general bone mineral outcomes (Table 1). Consequently, whether fetal neurocranial bones formed predominantly through intramembranous ossification represent a sensitive target of maternal ${\mathrm{PM}}_{2.5}$ exposure remains unknown. This gap is particularly relevant for populations chronically exposed to biomass-derived ${\mathrm{PM}}_{2.5}$, whose toxicological profile may induce placental dysfunction, hypoxia-responsive signaling, and impaired nutrient transport during critical windows of skeletal development. The interparietal and membranous occipital components may represent developmentally sensitive regions because they contribute to the posterior cranial fossa and follow ossification trajectories that differ from those of anterior cranial elements [15]. Therefore, posterior neurocranial mineralization may provide a sensitive morphological readout of systemic developmental perturbations.

We hypothesized that gestational exposure to wood-smoke-derived ${\mathrm{PM}}_{2.5}$ would delay fetal neurocranial mineralization and impair intramembranous bone maturation, and that these effects would be exacerbated when gestational exposure was preceded by pregestational exposure. We further hypothesized that these structural alterations would be associated with a hypoxia-responsive and osteogenic imbalance, reflected by altered HIF-1α, COL-I, and TGF-β immunoreactivity. Therefore, this study aimed to evaluate fetal growth, neurocranial mineralization, bone mineral density, histological maturation, and the immunohistochemical expression of osteogenic and hypoxia-related markers in a rat model of pregestational and gestational exposure to wood-smoke-derived ${\mathrm{PM}}_{2.5}$.

## 2. Results

During the experimental period, mean daily ambient concentrations of atmospheric pollutants were 48.8 ± 36.1 μ${\mathrm{g/m}}^{3}$ for ${\mathrm{PM}}_{2.5}$ (CV = 74.0%), 56.9 ± 38.3 μ${\mathrm{g/m}}^{3}$ for ${\mathrm{PM}}_{10}$ (CV = 67.3%), and 0.78 ± 0.49 ppm for CO (CV = 61.5%). The non-filtered air exposure chamber (NFA) recorded a mean ${\mathrm{PM}}_{2.5}$ concentration of 44.6 μ${\mathrm{g/m}}^{3}$, which was similar to the outdoor ambient concentration. In contrast, the filtered air chamber (FA) showed a significantly lower ${\mathrm{PM}}_{2.5}$ concentration of 3.0 ± 1.3 μ${\mathrm{g/m}}^{3}$ (CV = 34.3%; p<0.001), corresponding to a 94% reduction relative to the NFA chamber. The elevated ambient pollutant levels coincided with the austral winter, a period characterized by increased residential firewood combustion. Estimated cumulative inhaled ${\mathrm{PM}}_{2.5}$ mass during gestation was calculated using a minute ventilation rate of 0.043 L/min and an exposure duration of 21 days. This yielded an estimated inhaled air volume of 61.86 L/day and 1299 L, equivalent to 1.${\mathrm{299 m}}^{3}$, over the full gestational exposure period. Based on chamber ${\mathrm{PM}}_{2.5}$ concentrations, the estimated cumulative inhaled ${\mathrm{PM}}_{2.5}$ mass was 57.94 μg per female in the NFA condition and 3.90 μg per female in the FA condition (Supplementary Document S3).

### 2.1. Macroscopic Study

#### 2.1.1. Maternal Exposure to Wood-Smoke-Derived PM_2.5_ Was Associated with Reduced Fetal Growth

**Figure 1:**
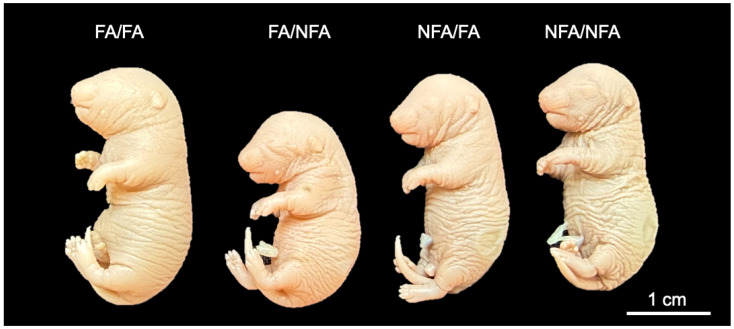
Representative gross morphology of rat fetuses at 21 days post-fertilization from the four experimental groups. Fetuses are shown in lateral view for FA/FA, FA/NFA, NFA/FA, and NFA/NFA exposure conditions. The first term indicates pregestational exposure and the second term indicates gestational exposure to either filtered air (FA) or non-filtered air (NFA). These representative specimens illustrate the fetal material used for morphometric, skeletal, radiographic, micro-CT, histological, and immunohistochemical analyses. Scale bar = 1 cm.

Maternal exposure history significantly modified fetal morphometric parameters at 21 days post-fertilization (dpf). For fetal body weight, two-way factorial ANOVA showed significant effects of pregestational exposure, gestational exposure, and their interaction (P: p=0.018; G: p<0.001; P × G: p=0.003). The FA/FA group showed the highest estimated marginal mean for fetal body weight (5.09 ± 0.14 g), whereas lower values were observed in the FA/NFA (4.11 ± 0.17 g), NFA/FA (4.61 ± 0.14 g), and NFA/NFA groups (4.53 ± 0.14 g). Relative to the FA/FA control group, fetal body weight was reduced by 19.4% in FA/NFA, 9.5% in NFA/FA, and 11.1% in NFA/NFA. Crown–rump length (CRL) was also significantly affected by pregestational and gestational exposure, although no significant interaction was detected (P: p=0.040; G: p=0.002; P × G: p=0.161). The FA/FA group showed the greatest CRL (39.25 ± 0.83 mm), whereas lower values were observed in FA/NFA (35.22 ± 0.96 mm), NFA/FA (36.96 ± 0.71 mm), and NFA/NFA (35.23 ± 0.71 mm). Relative to FA/FA, CRL was reduced by 10.3% in FA/NFA, 5.8% in NFA/FA, and 10.2% in NFA/NFA. Together, these findings indicate that pregestational and/or gestational exposure to wood-smoke-derived ${\mathrm{PM}}_{2.5}$ was associated with a reduced fetal size, as reflected by a lower fetal body weight and CRL compared with the filtered-air control condition Figure 1.

#### 2.1.2. Whole-Mount Skeletal Staining Revealed Delayed Neurocranial Mineralization

**Figure 2:**
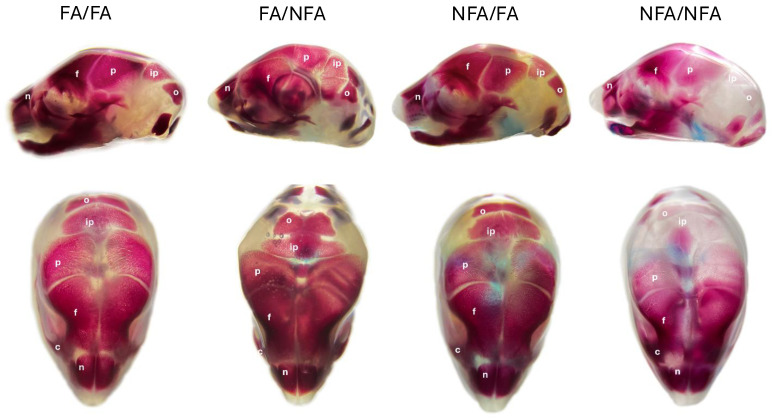
Clearing and alizarin red staining of fetal cranial bones at 21 days post-fertilization following pregestational and/or gestational exposure to wood-smoke-derived ${\mathrm{PM}}_{2.5}$. Representative cleared fetal skulls from the FA/FA, FA/NFA, NFA/FA and NFA/NFA experimental groups are shown in lateral and dorsal views. All groups exhibit positive alizarin red staining in the main bones of the neurocranium, confirming cranial mineralization at this developmental stage. The FA/FA group shows intense and relatively homogeneous staining, with well-delimited frontal, parietal, interparietal, occipital, nasal and zygomatic bones. In contrast, the NFA/NFA group displays weaker and less uniform staining, particularly in the interparietal and occipital regions, together with less clearly defined sutural boundaries and a broader anterior fontanelle. The FA/NFA and NFA/FA groups exhibit intermediate staining patterns, suggesting partial effects of gestational or pregestational ${\mathrm{PM}}_{2.5}$ exposure on membranous ossification and cranial suture development. Abbreviations: n, nasal bone; f, frontal bone; p, parietal bone; ip, interparietal bone; o, occipital bone; c, zygomatic bone.

Alizarin red staining confirmed the presence of mineralized bone tissue in all neurocranial elements across experimental groups (Figure 2). However, qualitative differences in staining intensity, uniformity and sutural morphology revealed a gradient of ossification maturity dependent on maternal ${\mathrm{PM}}_{2.5}$ exposure history. The FA/FA group exhibited the most advanced cranial development, characterized by intense homogeneous staining and narrow fontanelles with well-defined sutural boundaries. In contrast, NFA/NFA specimens displayed reduced staining intensity—most pronounced in interparietal and occipital bones—accompanied by poorly delimited sutures and a broader anterior fontanelle. Intermediate exposure groups (FA/NFA, NFA/FA) showed variable sutural definition and fontanelle closure patterns, suggesting temporal window-dependent effects. These findings indicate that continuous maternal ${\mathrm{PM}}_{2.5}$ exposure across pregestational and gestational periods disrupts intramembranous ossification progression and delays cranial suture maturation, with potential cumulative effects when exposure spans both developmental windows.

#### 2.1.3. Radiographic Assessment Confirmed Reduced Cranial Mineralization

**Figure 3:**
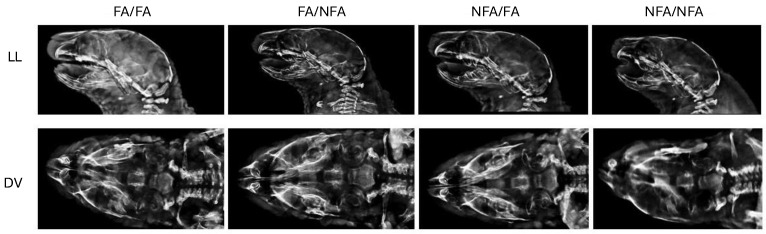
Radiographic evaluation of fetal cranial ossification at 21 days post-fertilization following pregestational and/or gestational exposure to wood-smoke-derived ${\mathrm{PM}}_{2.5}$. Representative radiographs of G3 fetal skulls from the FA/FA, FA/NFA, NFA/FA and NFA/NFA experimental groups are shown in latero-lateral (LL) and dorsoventral (DV) projections. The FA/FA group exhibits well-defined cranial bone contours and homogeneous mineralization of the neurocranium. In contrast, fetuses from the FA/NFA and NFA/FA groups show reduced radiographic density, less clearly defined frontoparietal and occipitotemporal regions and wider cranial sutures. The NFA/NFA group displays the most pronounced radiographic changes, characterized by diffuse mineralization, irregular cranial bone contours, and persistence of broad fontanelles, suggesting impaired intramembranous ossification associated with continuous maternal exposure to ${\mathrm{PM}}_{2.5}$.

Fetal cranial radiography in lateral and dorsoventral projections revealed a severity gradient dependent on maternal exposure pattern (Figure 3). In lateral view, the FA/FA group showed homogeneous neurocranial ossification, well-defined bone contours, and incipient sutural closure (sagittal/coronal). Gestational-only exposure (FA/NFA) produced frontoparietal radiolucencies, indistinct bone margins and persistently open sutures. Pregestational-only exposure (NFA/FA) induced irregular borders and occipitotemporal hypomineralization, evidencing osteogenic compromise prior to gestation. The NFA/NFA group exhibited the most severe pattern: diffuse mineralization defects, disrupted bone contours and persistently widened fontanelles. In dorsoventral projection, FA/FA maintained a homogeneous radiodensity in frontal and occipital regions. The FA/NFA and NFA/FA groups presented focal frontoparietal hypodensities, whereas NFA/NFA showed generalized osteopenia with loss of bone continuity throughout the neurocranium. Continuous exposure (NFA/NFA) generated the greatest structural deterioration, consistent with a potential cumulative effect of PM_2.5_ on cranial intramembranous ossification.

#### 2.1.4. Micro-CT Identified Region-Specific Mineral Density Deficits in Posterior Neurocranial Bones

**Figure 4:**
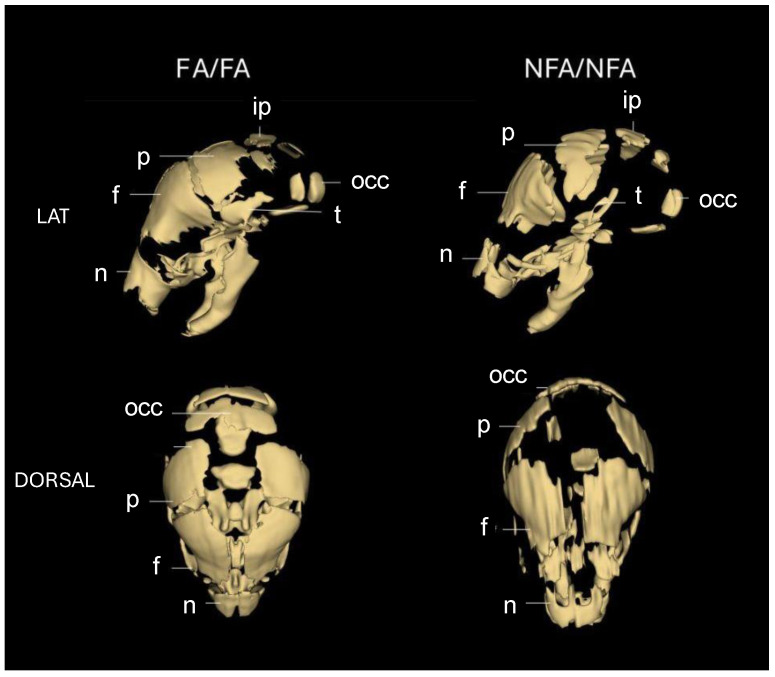
Micro-CT three-dimensional reconstruction of fetal cranial ossification at 21 days post-fertilization in the FA/FA and NFA/NFA experimental groups. Representative reconstructions are shown in lateral (LAT) and dorsal views. In the FA/FA group, the frontal (f), parietal (p), and nasal (n) bones exhibit a more continuous and symmetrical pattern of ossification, with well-defined bone contours and visible interosseous margins. The interparietal (ip), occipital (occ), and temporal (t) bones show incomplete mineralization, consistent with the developmental stage evaluated. In the NFA/NFA group, the frontal, parietal and nasal bones are identifiable; however, the interparietal and occipital bones exhibit reduced mineralization, irregular contours and discontinuity of the mineralized bone plate. These findings suggest that continuous maternal exposure to wood-smoke-derived ${\mathrm{PM}}_{2.5}$ during pregestational and gestational periods is associated with a topographically selective delay in fetal cranial ossification, particularly affecting the posterior neurocranial bones. Abbreviations: f, frontal bone; p, parietal bone; ip, interparietal bone; occ, occipital bone; t, temporal bone; n, nasal bone.

Three-dimensional micro-computed tomography analysis allowed qualitative and quantitative comparison of fetal neurocranial intramembranous ossification between the FA/FA and NFA/NFA groups, representing the two extremes of cumulative exposure (Figure 4). Nasal, frontal, parietal, interparietal, occipital, and temporal bones were identified in both groups, although with differences in developmental maturity and regional mineralization. In the FA/FA group, the frontal, parietal, and nasal bones presented symmetric and continuous ossification, with well-defined borders and visible interosseous margins. The interparietal and occipital bones showed areas of incomplete mineralization, consistent with the evaluated fetal developmental stage (GD21). In contrast, the NFA/NFA group exhibited ossification patterns comparable to controls in the frontal, nasal and parietal bones but showed reduced mineralization and irregular contours in the interparietal and occipital bones. This alteration was most pronounced in the interparietal bone, where reduced mineral density and discontinuity of the mineralized bone plate were observed. Temporal bones showed limited development in both groups, with more evident asymmetry in the spatial arrangement of the squamous portion in NFA/NFA fetuses. Zygomatic and maxillary bones were not visualized as completely formed structures in either group, consistent with the incomplete ossification expected at GD21. No gross disruption of cranial vault alignment or segmental discontinuity of mineralized structures was observed in either group.

Quantitative bone mineral density (BMD) analysis supported these qualitative observations in the regions selected for measurement (Table 2). Interparietal and occipital bones exhibited significant reductions in BMD in NFA/NFA compared with FA/FA fetuses (20.8% and 20.2%, respectively; p=0.0086 and p=0.0123), whereas frontal and parietal bones showed only minor, non-significant differences between groups (3.6% and 4.1%, respectively; p>0.05). Thus, quantitative micro-CT identified a topographically selective mineralization deficit affecting posterior neurocranial bones under continuous maternal PM_2.5_ exposure. Quantitative BMD analysis was intentionally restricted to the FA/FA and NFA/NFA groups for three pre-specified reasons. First, these two groups represent the extreme conditions of cumulative PM_2.5_ exposure, thereby maximizing biological contrast and statistical power to detect region-specific mineralization deficits. Second, qualitative assessment across all four groups identified the interparietal and occipital bones as the most susceptible neurocranial regions, and concentrating quantitative BMD measurement on the maximal-contrast comparison enhanced detection sensitivity for these site-specific effects. Third, micro-CT scanning and quantitative BMD analysis are resource-intensive procedures; restricting quantitative analysis to the two extreme groups optimized analytical resources while providing the highest-quality evidence for the primary comparison. It is explicitly acknowledged that this decision precludes a formal estimation of independent pregestational effects, gestational effects, or pregestational × gestational interaction effects on BMD; such decomposition would require quantitative micro-CT analysis of all four groups and represents a methodological refinement for future studies. Inferences regarding exposure-window specificity were therefore derived from the four-group morphometric, radiographic, whole-mount staining, histological, and immunohistochemical analyses, whereas micro-CT provided high-resolution regional confirmation of the mineralization phenotype in the maximal-contrast comparison.

Bone mineral density (BMD) values are expressed as mean ± standard deviation in mg hydroxyapatite (HA)/cm^3^. Five ROI measurements per fetus were averaged to yield one BMD value per fetus per bone (technical replicates; not used as independent observations). Statistical comparisons were performed on fetal-level means. Biological *n*: FA/FA =5 fetuses; NFA/NFA =5 fetuses (n=25 ROI measurements per group represent technical replicates only and are reported for transparency).

### 2.2. Microscopic Study

#### 2.2.1. Histology Showed Delayed Maturation of Intramembranous Bone Tissue

Histological evaluation of fetal parietal bone sections revealed notable differences in immature bone tissue (*textus osseus immaturus*) maturation according to maternal ${\mathrm{PM}}_{2.5}$ exposure history. The FA/FA group exhibited well-defined trabecular organization, homogeneous matrix acidophilia, preserved structural margins and minimal osteoid (*osteoidum*), consistent with advanced intramembranous ossification. Osteocytes (*osteocyti*) were regularly distributed within bone lacunae, osteoprogenitor cells (*cellulae osteogenicae*) were observed at the trabecular periphery, and osteoblasts (*osteoblasti*) were sparse and mainly localized along the peripheral border of developing bone tissue. In contrast, NFA/NFA specimens showed reduced matrix acidophilia, irregular trabecular margins, abundant osteoid and limited osteocyte incorporation into bone lacunae. Osteogenic cells were less evident at the trabecular periphery, whereas osteoblasts were numerous and diffusely distributed along trabecular surfaces, indicating delayed tissue maturation and persistence of osteoblast-lined bone surfaces. Intermediate exposure groups displayed transitional histological patterns: FA/NFA resembled FA/FA but showed reduced osteocyte density and low osteoblast density predominantly in the mid-region, whereas NFA/FA shared features with NFA/NFA but exhibited higher matrix acidophilia, better-defined trabecular contours and intermediate osteoblast density with broad peripheral distribution.

#### 2.2.2. Immunohistochemistry Revealed Marker-Specific Differences in Fetal Parietal Bone

**Figure 5:**
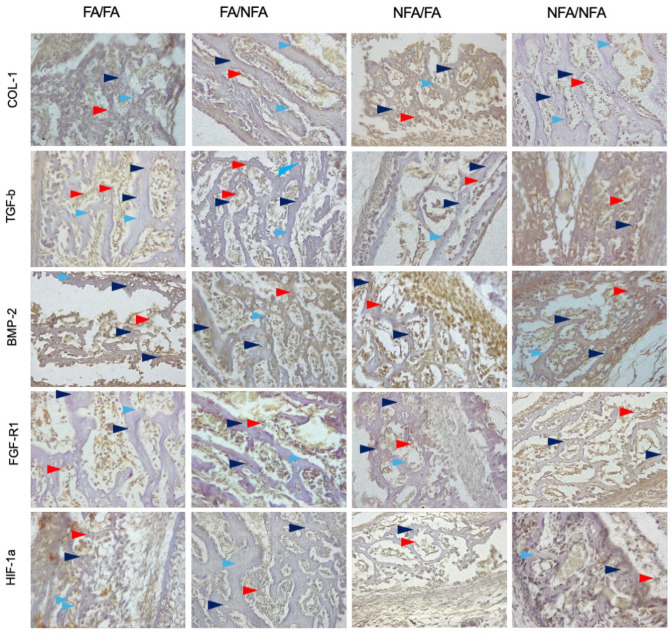
Representative immunohistochemical staining of osteogenic and hypoxia-related markers in fetal parietal bone. Immunoreactivity for COL-1, TGF-β, BMP-2, FGF-R1, and HIF-1α is shown as brown DAB signal in the developing cranial vault across the FA/FA, FA/NFA, NFA/FA, and NFA/NFA experimental groups. Red arrowheads indicate osteoblast-like cells located along developing bone surfaces, dark blue arrowheads indicate osteocytes located within bone lacunae, and light blue arrowheads indicate trabecular bone margins. Sections were counterstained with hematoxylin to visualize nuclei. Magnification: 40×.

**Figure 6:**
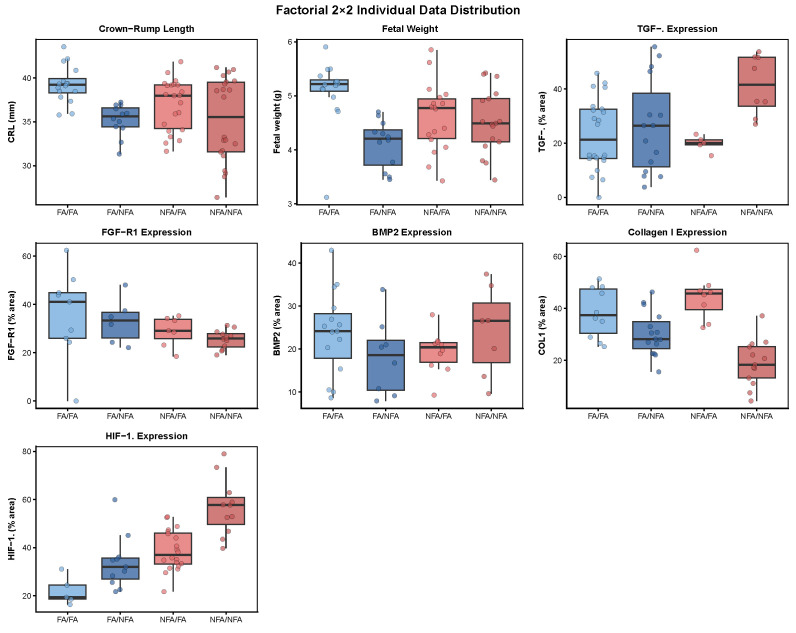
Distribution of fetal morphometric and immunohistochemical outcomes according to maternal ${\mathrm{PM}}_{2.5}$ exposure history. Boxplots with individual data points show crown–rump length (CRL, mm), fetal body weight (g), and immunoreactive area fraction (%) for HIF-1α, COL1, TGF-β, BMP2, and FGF-R1 in fetal parietal bone at 21 days post-fertilization. Box elements indicate the median, interquartile range (IQR), and whiskers extending to 1.5× IQR. Exposure groups: FA/FA, filtered air during pregestation and gestation; FA/NFA, filtered air during pregestation and non-filtered air during gestation; NFA/FA, non-filtered air during pregestation and filtered air during gestation; NFA/NFA, non-filtered air during pregestation and gestation. Factorial ANOVA results are summarized in Table 3. Statistical group comparisons (Tukey HSD post hoc results) are reported in Table 3 and are not superimposed on the figure to avoid visual redundancy.

Quantitative immunohistochemical analysis of fetal parietal bone revealed marker-specific differences among experimental groups (Table 3; Figure 5 and Figure 6). Two-way factorial ANOVA showed significant main effects of pregestational and gestational exposure on HIF-1α immunoreactivity (P: p=0.001; G: p=0.030), with no significant interaction effect (P × G: p=0.334). COL1 immunoreactivity showed a significant gestational effect (p=0.032) and a significant pregestational × gestational interaction (p=0.003), whereas the pregestational effect was not significant (p=0.151). TGF-β immunoreactivity showed a significant interaction effect (p=0.049), with no significant main effects of pregestational or gestational exposure (p=0.624 and p=0.502, respectively). In contrast, BMP2 and FGF-R1 showed no significant main effects or interaction effects (p>0.05 for all factorial terms).

Estimated marginal means showed that HIF-1α immunoreactivity was lowest in FA/FA and highest in NFA/NFA. COL1 immunoreactivity showed an interaction-dependent pattern, with the lowest value observed in NFA/NFA and the highest value in NFA/FA. TGF-β immunoreactivity was highest in NFA/NFA and lowest in NFA/FA. BMP2 and FGF-R1 showed comparatively similar values across groups, consistent with the absence of significant factorial effects.

#### 2.2.3. Integrated Morphological and Molecular Features of the Altered Ossification Phenotype

Histological and immunohistochemical findings converged on an altered maturation pattern of intramembranous bone tissue in exposed groups, particularly in NFA/NFA fetuses. In this group, the parietal bone showed less homogeneous matrix staining, abundant osteoid, irregular trabecular margins, reduced osteocyte incorporation, and broadly distributed osteoblasts along developing bone surfaces. This histological pattern coincided with increased HIF-1α and TGF-β immunoreactivity and reduced COL1 immunoreactivity relative to the FA/FA group. Together, these findings suggest that continuous exposure to wood-smoke-derived ${\mathrm{PM}}_{2.5}$ was associated with delayed matrix maturation and altered expression of markers related to hypoxia-associated stress, extracellular matrix organization, and osteogenic signaling in fetal intramembranous bone tissue.

## 3. Discussion

The present findings should be interpreted primarily as evidence of altered fetal neurocranial intramembranous ossification rather than as a generalized skeletal phenotype. The most consistent alterations were detected in membranous cranial bones, particularly the interparietal and occipital regions, where reduced mineral density, weaker alizarin red staining, less advanced sutural narrowing, and histological features consistent with delayed matrix maturation were observed. Because endochondral skeletal elements were not systematically quantified in the present study, conclusions regarding PM_2.5_-associated skeletal toxicity should be restricted to membranous neurocranial ossification.

### 3.1. ${\mathrm{PM}}_{2.5}$ Exposure Was Associated with Reduced Fetal Growth and Neurocranial Mineralization

The observed reductions in fetal weight, crown–rump length, and craniofacial mineralization across ${\mathrm{PM}}_{2.5}$-exposed groups indicate that wood-smoke-derived particulate exposure was associated with altered fetal development in this experimental model. Fetal growth restriction associated with ambient ${\mathrm{PM}}_{2.5}$ exposure has been consistently described in human cohorts [17] and in murine models exposed to particulate matter [19] and is now reproducibly reported for biomass-derived ${\mathrm{PM}}_{2.5}$ in the Sprague–Dawley model employed here [9]. Within this broader framework, our morphometric findings suggest that the observed neurocranial skeletal phenotype may occur in parallel with broader placental and metabolic perturbations affecting intrauterine homeostasis. Previous studies from our laboratory using this experimental model demonstrated that gestational ${\mathrm{PM}}_{2.5}$ exposure induced placental adaptations, including reduced theoretical and specific oxygen diffusion capacity, upregulation of HIF-1α and VEGF expression, downregulation of GLUT1, and compensatory remodeling of SVCT2 [9,10]. While these placental parameters were not assessed in the current study, such alterations provide a plausible biological context in which oxygen, glucose, and ascorbate availability to the developing fetus could be affected. In humans, maternal ${\mathrm{PM}}_{2.5}$ exposure during pregnancy has been associated with deteriorated maternal trabecular and cortical bone strength [13], suggesting broader skeletal impacts during the perinatal period. Black-carbon nanoparticles have been visualized on the fetal side of human placentas [3], indicating that direct fetal contact with combustion-derived particles is biologically plausible, although particle translocation was not evaluated in the present study. Bone formation requires coordinated supply of oxygen, glucose, and ascorbate, the latter serving as a cofactor for prolyl- and lysyl-hydroxylation during collagen synthesis [20]. Our finding that BMD reductions were concentrated in the interparietal and occipital bones, whereas frontal and parietal regions showed only minor non-significant differences, suggests differential vulnerability of posterior neurocranial structures to ${\mathrm{PM}}_{2.5}$ exposure. This topographic selectivity argues against a merely generalized reduction in fetal size and instead supports the presence of a region-specific delay in neurocranial mineralization. The observed craniofacial deficits therefore integrate placental, vascular, and metabolic perturbations into quantifiable structural readouts, including mineralization density, sutural morphology, and bone contour integrity. This framework aligns with the developmental origins of health and disease (DOHaD) paradigm, in which environmental exposures during sensitive windows of organogenesis can leave lasting structural signatures on the offspring [21,22]. Whether these craniofacial alterations persist postnatally, normalize after birth, or predispose to long-term skeletal fragility warrants investigation in longitudinal follow-up studies.

### 3.2. Histological and Immunohistochemical Evidence of Delayed Osteogenic Maturation and Altered Matrix Organization

Histological evaluation of the fetal parietal bone provided cellular-level support for the radiographic and tomographic findings. Across exposed groups, most evidently in NFA/NFA fetuses, developing bone tissue exhibited less homogeneous matrix staining, irregular margins, increased osteoid-like matrix, and reduced osteocyte incorporation into lacunae, while osteoblast-like cells were numerous and broadly distributed across trabecular surfaces rather than being confined to discrete bone-forming fronts. In control fetuses, osteoblast-like cells were mainly peripheral and less abundant, consistent with a more advanced maturation pattern of intramembranous bone tissue. In contrast, NFA/NFA fetuses exhibited broad osteoblast-like cell distribution with persistent osteoid-like matrix and reduced osteocyte density, compatible with delayed matrix maturation. The osteoblast-to-osteocyte transition has been described as a maturation-dependent process involving progressive cellular embedding within the mineralizing matrix [23]; however, this process was not directly evaluated in the present study [23]. Therefore, the observed spatial distribution of osteoblast-like cells should be interpreted as a morphological indicator of delayed tissue maturation, warranting future evaluation using osteocyte markers such as DMP-1 and sclerostin. Immunohistochemical analysis revealed marker-specific alterations involving reduced COL1 immunoreactivity in association with reduced mineralization. COL1 immunoreactivity was approximately 50% lower in NFA/NFA fetuses than in FA/FA controls, with weaker and more discontinuous labeling associated with reduced matrix organization and irregular osteoid-like areas. This pattern is consistent with altered collagen matrix organization, although it should not be interpreted as a direct quantitative measurement of total collagen content. Reduced type I collagen immunoreactivity under environmental stress has been reported in other exposure models, where it accompanies deficient mineralization [24]. Notably, the NFA/FA group exhibited the highest COL-1 expression among all groups, significantly exceeding both FA/NFA (p=0.0038) and NFA/NFA (p<0.0001), and even surpassing FA/FA controls, suggesting a distinct COL1 immunoreactivity pattern. Whether this reflects compensatory regulation requires further investigation. The immunohistochemical profile provides context for interpreting this mineralization-limited phenotype. HIF-1α immunoreactivity differed among exposure groups, with the highest values in NFA/NFA fetuses (2.6-fold vs. FA/FA, p<0.0001), showing extensive diffuse labeling throughout osteogenic compartments. This pattern supports a hypoxia-associated or stress-responsive profile within developing bone tissue, but should not be interpreted as direct evidence of reduced oxygen tension. While HIF-1α can promote osteoblast differentiation and bone formation under certain conditions [25,26], sustained elevation in the context of environmental pollutant exposure may be associated with impaired osteogenic maturation [27]. This HIF-1α-positive profile coincided with higher TGF-β immunoreactivity in NFA/NFA fetuses (1.8-fold vs. FA/FA, p=0.0144), in the context of a significant pregestational × gestational interaction potentially relevant to the altered matrix phenotype. Indeed, ${\mathrm{PM}}_{2.5}$ exposure has been shown to affect osteoblast differentiation through TGF-β-related signaling, including Smad4 degradation and reduced expression of osteogenic transcription factors and matrix proteins [28]; however, these downstream targets were not evaluated in the present study. Importantly, BMP-2 and FGF-R1 immunoreactivity did not differ significantly across groups (p>0.05), suggesting that maternal ${\mathrm{PM}}_{2.5}$ exposure selectively altered HIF-1α, TGF-β, and COL1 immunoreactivity rather than uniformly affecting all osteogenic markers evaluated. These findings support a phenotype characterized by reduced mineral deposition, lower COL1 immunoreactivity, increased HIF-1α immunoreactivity, and altered matrix organization. Therefore, the potential involvement of hypoxia-associated signaling, TGF-β-related pathways, altered osteoblast activity, and collagen gene regulation remains inferential and requires direct experimental validation.

### 3.3. Hypoxia-Associated Pathways as Plausible Mediators of Delayed Intramembranous Ossification

The immunohistochemical profile observed in fetal parietal bone suggests a stress-responsive osteogenic microenvironment involving hypoxia-associated signaling and altered matrix maturation. HIF-1α immunoreactivity was increased in NFA/NFA fetuses compared with FA/FA controls (2.6-fold), consistent with the significant effects of pregestational and gestational exposure detected in the factorial analysis. This pattern parallels the hypoxia-related response previously described in the placental compartment [9] and is biologically consistent with the role of HIF-1α in coordinating angiogenic and osteogenic processes during skeletal development [26,27]. However, increased HIF-1α immunoreactivity should not be interpreted as direct evidence of reduced fetal bone oxygen tension, because HIF-1α stabilization may also occur in response to oxidative stress, inflammation, or metabolic dysregulation. Therefore, the relationship between maternal PM_2.5_ exposure, placental dysfunction, fetal hypoxia-associated signaling, and delayed intramembranous ossification should be interpreted as a biologically plausible pathway supported by convergent tissue-level evidence rather than as a causally demonstrated mechanism.

Increased HIF-1α immunoreactivity at 21 dpf may indicate an adaptive or stress-associated response in developing osteogenic tissue. Although transient HIF-1α activation can contribute to vascular invasion, osteoprogenitor recruitment, and bone formation, prolonged or dysregulated activation has been associated with impaired osteoblast maturation and reduced mineralization under pathological conditions [25,27]. In the present study, the HIF-1α-positive profile coincided with reduced COL1 immunoreactivity in NFA/NFA fetuses, supporting the interpretation that continuous exposure was associated with altered collagen matrix maturation. This is relevant because collagen-I constitutes the principal organic scaffold for mineral deposition during intramembranous ossification; therefore, reduced or discontinuous COL1 immunoreactivity provides a plausible histomolecular correlate for the mineralization deficits observed by micro-CT and histological assessment.

TGF-β immunoreactivity showed a significant pregestational × gestational interaction, indicating that its expression pattern was not explained by either exposure window alone. Although the NFA/NFA group presented the highest mean TGF-β immunoreactivity, this pattern should be interpreted cautiously according to the post hoc structure of estimated marginal means. Rather than indicating a uniform increase relative to controls, the data suggest exposure-window-dependent modulation of TGF-β signaling. This finding may be biologically relevant because experimental evidence indicates that PM_2.5_ exposure can alter TGF-β signaling and affect osteogenic differentiation through pathways involving Smad regulation, Runx2, Sp7, and downstream matrix proteins such as collagen-I [28]. Thus, altered TGF-β signaling may provide a plausible regulatory context for the COL1 reduction observed under continuous exposure, but this mechanism remains inferential because Smad activity, osteogenic transcription factors, and collagen gene expression were not directly assessed in the present study.

In contrast, BMP2 and FGF-R1 showed no significant main or interaction effects, suggesting that maternal PM_2.5_ exposure did not uniformly modify all osteogenic markers evaluated. The delayed ossification phenotype appeared more closely associated with stress-responsive signaling and collagen matrix alteration, particularly involving HIF-1α, TGF-β, and COL1, than with broad suppression of BMP2 or FGF-R1 immunoreactivity. However, this should not be interpreted as definitive evidence that canonical osteogenic signaling was unaffected, because only selected markers were evaluated at a single developmental time point.

Overall, the immunohistochemical findings suggest that continuous maternal exposure to wood-smoke-derived PM_2.5_ was associated with increased HIF-1α immunoreactivity, exposure-window-dependent modulation of TGF-β, reduced COL1 immunoreactivity, and deficient mineralization of fetal intramembranous bone. This phenotype is consistent with epidemiological and Mendelian randomization evidence linking PM_2.5_ exposure to lower bone mineral density [14,29] and supports inflammatory, oxidative, and hypoxia-associated pathways as plausible contributors to skeletal vulnerability. However, the PM_2.5_–placenta–hypoxia-associated signaling–ossification axis remains a mechanistic hypothesis requiring further validation.

### 3.4. Pregestational Exposure and Developmental Programming

A distinctive contribution of this work is that it suggests a contribution of pregestational ${\mathrm{PM}}_{2.5}$ exposure to fetal skeletal development, even when gestation occurs under filtered-air conditions. Most studies addressing ${\mathrm{PM}}_{2.5}$ and fetal development focus on gestational exposure as the primary window of susceptibility [17], implicitly giving less attention to the biological relevance of the pre-conception maternal environment. Our findings are consistent with emerging evidence indicating that the periconceptional environment may influence oocyte quality, uterine receptivity, and early developmental programming of the conceptus [30,31,32]. Several non-mutually exclusive mechanisms may account for this pattern. First, ${\mathrm{PM}}_{2.5}$-induced oxidative stress and endocrine disruption during the pregestational period may alter follicular dynamics and steroidogenesis, as documented in this model [11], potentially influencing early embryonic programming and mesenchymal commitment toward the osteoblastic lineage. Second, persistent uteroplacental alterations following pregestational ${\mathrm{PM}}_{2.5}$ exposure [9] may compromise nutrient and oxygen supply during subsequent gestation, even when the gestational atmosphere is filtered. Third, environmentally induced epigenetic modifications in maternal somatic or germ cells before conception may provide a biological substrate for pre-conception environmental memory; air-pollution-related changes in DNA methylation and chromatin accessibility have been increasingly documented [30,31]. These findings align with the developmental origins of health and disease (DOHaD) framework and suggest that interventions restricted exclusively to gestation may be insufficient to fully restore fetal skeletal development when the pre-conception maternal environment has been compromised. Nevertheless, this interpretation remains inferential because oocyte quality, uterine receptivity, placental function, and epigenetic profiles were not directly evaluated in the present study. Direct demonstration of pre-conception programming or intergenerational transmission will require methylome and transcriptome profiling of oocytes, placentas, and fetal skeletal tissues, representing an important avenue for future work.

### 3.5. Methodological Strengths and Limitations

This study advances the field through several methodological strengths. First, exposure was performed in environmentally relevant atmospheric chambers that captured the chemical complexity of biomass-derived PM_2.5_ at concentrations characteristic of southern Chilean cities during the austral winter. Second, the multigenerational exposure design (G1 → G2) integrated pregestational and gestational exposure windows, allowing assessment of exposure patterns that are rarely examined in airborne-pollution studies. Third, the multiscale phenotyping strategy, integrating fetal morphometry, radiography, micro-CT, whole-mount skeletal staining, histology, and immunohistochemistry [33,34], provided convergent evidence across complementary anatomical, structural, and molecular levels. Finally, blinded analysis, intraclass correlation validation, and transparent reporting strengthened the reliability and reproducibility of the observations. These strengths should be interpreted alongside several limitations. First, transcriptomic, epigenomic, and metabolomic profiling was not performed; therefore, the observed alterations in matrix organization and mineralization, as well as the inferred role of hypoxia-related developmental programming, remain biologically plausible but not directly demonstrated [35]. In addition, direct measurements of fetal tissue oxygen tension, oxidative stress, local vascularization, and osteoblast functional activity were not performed. Consequently, the proposed PM_2.5_–placenta–hypoxia-associated signaling–ossification axis should be regarded as an inferential mechanistic model requiring further experimental validation. Second, biomechanical testing of fetal craniofacial bones was not conducted, precluding assessment of the functional consequences of the observed mineralization deficits [33]. Third, elemental analysis of mineralized matrices, including calcium-to-phosphorus ratios and trace metal accumulation, was not performed. Fourth, the immunohistochemical sample size may have limited the statistical power to detect subtle changes in BMP-2 and FGF-R1 immunoreactivity. Fifth, histological assessment of the parietal bone (Section 4.7.1) was qualitative/descriptive and was not complemented by quantitative histomorphometry, such as osteoid thickness, osteocyte or osteoblast density, or mineralized-to-non-mineralized matrix ratio. Incorporating such measurements in future studies would provide a quantitative structural correlate to the immunohistochemical and micro-CT findings. Sixth, postnatal follow-up was not undertaken; therefore, whether the observed delay in intramembranous ossification resolves through catch-up mineralization or persists into postnatal life remains unknown and clinically relevant [36]. An additional methodological consideration concerns the hierarchical structure of fetal developmental data; the sampling design adopted to address intra-litter dependence is detailed in Section 4.8.

### 3.6. Biological and Public-Health Implications

Integrating placental, vascular, hormonal, and skeletal evidence from this experimental program [9,10,11] supports a coherent interpretation: maternal exposure to wood-smoke-derived ${\mathrm{PM}}_{2.5}$ may configure an intrauterine environment characterized by hypoxia-associated signaling, redox imbalance, and altered nutrient availability, within which the fetal skeleton develops structural and molecular signatures consistent with delayed maturation. The present findings situate the neurocranium, particularly the occipital and interparietal compartments, as a sensitive anatomical substrate for the effects of biomass-derived air pollution on prenatal development [21]. They also provide experimental correlates for epidemiological evidence linking ambient ${\mathrm{PM}}_{2.5}$ exposure with reduced fetal biometry [17], increased risk of craniofacial and structural anomalies [37,38], and developmental programming across the life course [22]. From a public-health perspective, residential wood combustion is a major contributor to wintertime ${\mathrm{PM}}_{2.5}$ pollution in southern Chile and in many low- and middle-income settings where particulate concentrations frequently exceed WHO guidelines. Demonstrating that both gestational and pregestational exposure to this particulate signature are associated with altered fetal intramembranous ossification reframes wood-smoke pollution as a developmental risk with potential life-course implications for skeletal health [14,21,29]. The morphological readouts characterized here—mineralization density, osteoid-like matrix organization, and regional neurocranial heterogeneity—may represent candidate fetal indicators of prenatal environmental insult and could inform future translational studies using prenatal imaging, postnatal craniofacial morphometry, and longitudinal skeletal assessment. Overall, continuous maternal exposure to wood-smoke-derived ${\mathrm{PM}}_{2.5}$ was associated with alterations in the fetal osteogenic microenvironment and delayed intramembranous ossification, plausibly involving hypoxia-associated signaling, altered extracellular matrix maturation, and persistence of immature osteogenic features. These findings provide a morphological foundation for considering prenatal protection from biomass-derived particulate matter as a relevant priority in maternal–fetal medicine and environmental public-health policy, particularly in vulnerable socioenvironmental contexts.

## 4. Materials and Methods

### 4.1. Study Site and Environmental Context

This study was conducted in Temuco, southern Chile (38°44′59.4″ S, 72°37′07.8″ W), an urban area with persistently elevated concentrations of fine particulate matter (PM_2.5_) (Supplementary Document S1). Residential wood combustion for domestic heating represents the principal emission source, contributing to severe wintertime air pollution episodes. In 2021, Temuco ranked among the cities with the highest annual PM_2.5_ concentrations globally according to the IQAir World Air Quality Report [39]. The experimental exposure period extended from 15 June to 30 September 2021 (austral winter), coinciding with peak residential biomass combustion activity and maximum atmospheric PM_2.5_ concentrations in the region (Supplementary Document S2).

### 4.2. Exposure System Design and Operation

**Figure 7:**
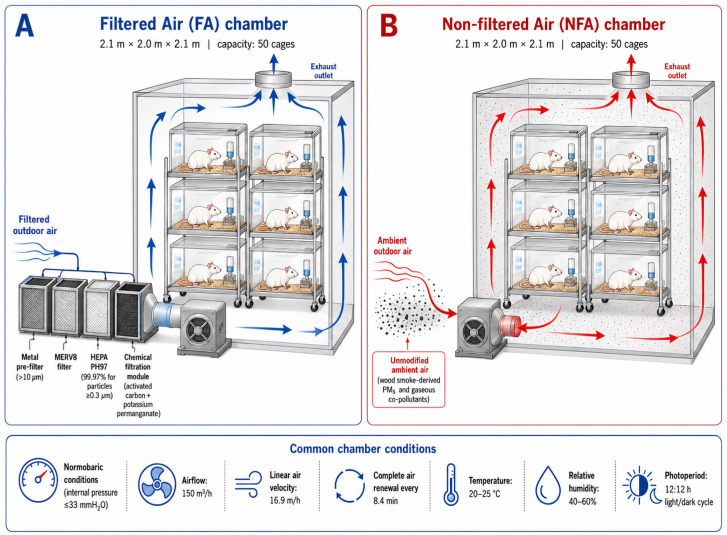
Exposure chamber configuration for filtered air and non-filtered ambient air conditions. (**A**) Filtered air (FA) chamber used to generate clean-air control conditions. Ambient outdoor air was drawn into the system and passed sequentially through a metallic pre-filter for coarse particle retention (>10 μm), a pleated MERV8 medium-efficiency filter, a HEPA PH97 filter with 99.97% retention efficiency for particles ≥0.3 μm, and a chemical filtration module containing activated carbon and potassium permanganate-impregnated media. Filtered air was introduced into the chamber by an industrial centrifugal fan and distributed throughout the chamber before being exhausted through a superior outlet. (**B**) Non-filtered air (NFA) chamber used to reproduce real-world ambient exposure conditions. In that chamber, outdoor air from an urban environment characterized by wood-smoke-derived ${\mathrm{PM}}_{2.5}$ was introduced without filtration, allowing exposure to fine particulate matter and associated gaseous co-pollutants. Both chambers had identical dimensions of 2.1 m × 2.0 m × 2.1 m and a maximum capacity of 50 standardized animal cages. The system operated under normobaric conditions, with an airflow of $150\;m^{3}$/h, linear air velocity of 16.9 m/h, and complete air renewal every 8.4 min. Temperature, relative humidity, and photoperiod were maintained at 20–25 °C, 40–60%, and 12:12 h light/dark cycle, respectively. This schematic was generated with AI assistance (ChatGPT, OpenAI, GPT-5.3); all outputs were critically reviewed and validated by the authors.

The experimental exposure system comprised two adjacent chambers designed to provide controlled environmental conditions for animal housing during ambient air exposure [40,41,42]. This system allowed the simulation of environmentally relevant differences in particulate matter concentration under controlled conditions. Each chamber measured 2.1 m × 2.0 m × 2.1 m (height × width × depth) with capacity for 50 standardized animal cages. The chambers operated under normobaric conditions (internal pressure ≤33 mmH_2_O) to maintain physiological equivalence with ambient atmospheric pressure. Continuous airflow into each chamber was generated by an industrial centrifugal fan (model CBB60N, Zepol S.L., Madrid, Spain) positioned at the chamber base, operating at 150 m^3^/h with linear air velocity of 16.9 m/h. This configuration ensured complete air renewal every 8.4 min (total chamber volume: 21 m^3^; flow rate: 2.5 m^3^/min), promoting homogeneous distribution throughout the vertical axis before exhaust through a superior outlet. Environmental air was drawn from an urban monitoring site characterized by elevated wood-smoke pollution levels. The filtered air (FA) chamber incorporated a three-stage sequential filtration system to generate clean-air control conditions: (1) a metallic pre-filter for retention of coarse particles (>10 μm); (2) a pleated MERV8 medium-efficiency filter (ASHRAE 52.2 standard); and (3) a high-efficiency particulate air (HEPA) filter PH97 (99.97% retention efficiency for particles ≥0.3 μm). Additionally, a chemical filtration module (Purafil PSA 102, 500 cfm; Purafil Inc., Doraville, GA, USA) equipped with activated carbon and potassium permanganate impregnated media (Purafil Select PK12) was integrated upstream to reduce gaseous pollutants and volatile organic compounds. This multi-stage filtration configuration has been validated in previous controlled exposure studies [11,41]. The non-filtered air (NFA) chamber received unmodified ambient air without filtration, representing real-world urban exposure conditions. This configuration allowed direct inhalation of wood-smoke-derived PM_2.5_ and associated gaseous co-pollutants present in the urban atmosphere during the winter heating season (Figure 7). Both chambers maintained equivalent temperature (20–25 °C), relative humidity (40–60%), and photoperiod (12:12 h light/dark cycle) throughout the experimental period.

### 4.3. Environmental Monitoring and Air Quality Assessment

Fine particulate matter (${\mathrm{PM}}_{2.5}$) concentrations were continuously monitored in both exposure chambers using automated gravimetric measurement systems. Outdoor ambient ${\mathrm{PM}}_{2.5}$ concentrations were determined using a beta-attenuation monitor (BAM 1020; Met One Instruments Inc., Grant Pass, OR, USA) equipped with a carbon-14 beta-radiation source (60 μCi ± 15 μCi) and scintillation detector, operating at constant airflow of 16.7 L/min under standardized reference conditions [43]. This method provides real-time mass concentration measurements (μ${\mathrm{g/m}}^{3}$) based on beta-ray attenuation proportional to particle deposition on collection filters [44]. Complementary air quality indicators included carbon monoxide (CO) and nitrogen dioxide (${\mathrm{NO}}_{2}$) concentrations. CO concentrations were quantified as 8 h moving averages by non-dispersive infrared spectroscopy (Teledyne T300), while ${\mathrm{NO}}_{2}$ was measured as 24 h averages using gas-phase chemiluminescence (Thermo Scientific 42i, Thermo Fisher Scientific, Waltham, MA, USA). Environmental measurements were obtained from the “Las Encinas Air Quality Monitoring Station” (Algoritmos y Mediciones Ambientales SpA, Santiago, Chile), located 200 m from the exposure site. All monitoring data underwent quality assurance validation and were publicly reported through the Chilean National Air Quality Information System (SINCA; https://sinca.mma.gob.cl, accessed on 30 October 2021). The filtration system in the FA chamber effectively reduced particulate matter concentrations but was not designed to eliminate all gaseous pollutants. Consequently, CO and ${\mathrm{NO}}_{2}$ concentrations remained comparable between FA and NFA chambers throughout the exposure period, reflecting background urban gaseous pollution levels common to both environments [9,11]. Chamber-specific environmental parameters, including daily ${\mathrm{PM}}_{2.5}$ and ${\mathrm{PM}}_{10}$ concentrations, CO and ${\mathrm{NO}}_{2}$ 8 h averages, temperature, and relative humidity recorded throughout the exposure period, are summarized in Supplementary Document S2. CO and ${\mathrm{NO}}_{2}$ concentrations were comparable between FA and NFA chambers, reflecting background urban gaseous pollution common to both environments, as the filtration system was designed for particulate removal and not gaseous pollutant elimination. Particle-size distribution was not directly characterized in the exposure chambers; this is acknowledged as a limitation. Chemical speciation of ${\mathrm{PM}}_{2.5}$ in the NFA chamber was not performed; the exposed animals inhaled the ambient wood-smoke-derived aerosol mixture as it occurred under real-world wintertime conditions in Temuco.

### 4.4. Experimental Design and Animal Allocation

#### 4.4.1. Multigenerational Exposure Protocol

**Figure 8:**
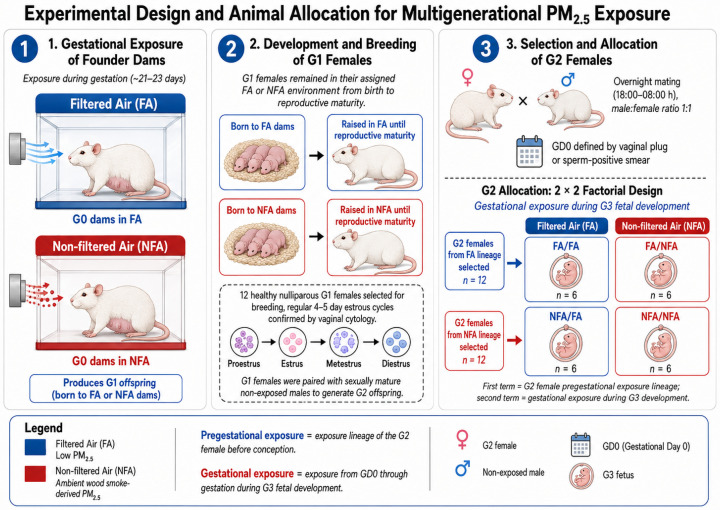
Experimental design and animal allocation for the multigenerational ${\mathrm{PM}}_{2.5}$ exposure model. (1) Gestational exposure of founder dams. Pregnant Sprague–Dawley rats were housed during gestation in either filtered air (FA; low ${\mathrm{PM}}_{2.5}$) or non-filtered air (NFA; ambient wood-smoke-derived ${\mathrm{PM}}_{2.5}$) chambers, generating G1 offspring. (2) Development and breeding of G1 females. Female G1 offspring born from FA- or NFA-exposed dams remained under their assigned environmental condition from birth until reproductive maturity. Twelve healthy nulliparous G1 females exhibiting regular 4–5 day estrous cycles, confirmed by vaginal cytology, were selected for breeding and paired with sexually mature non-exposed males to generate G2 offspring. (3) Selection and allocation of G2 females. Twelve healthy nulliparous G2 females were selected for the experimental pregnancy protocol and paired overnight with sexually mature non-exposed males at a 1:1 male:female ratio. Gestational day 0 (GD0) was defined by the detection of a vaginal copulation plug or sperm-positive vaginal smear. Pregnant G2 females were allocated according to a 2 × 2 factorial design based on pregestational exposure lineage and gestational exposure condition, generating four experimental groups: FA/FA, FA/NFA, NFA/FA, and NFA/NFA. The first term indicates the pregestational exposure lineage of the G2 female, whereas the second term indicates the gestational exposure condition during G3 fetal development. Six G3 fetuses per group were evaluated. Arrows indicate the chronological progression of exposure, breeding, gestation and fetal evaluation across generations. This schematic was generated with AI assistance (ChatGPT, OpenAI, GPT-5.3); all outputs were critically reviewed and validated by the authors.

The study protocol received approval from the Scientific Ethics Committee of Universidad de La Frontera (Record No. 122/20) and was conducted in accordance with Chilean Law No. 20.380 on Animal Protection and the ARRIVE 2.0 guidelines for transparent reporting of animal research [45]. The experimental design employed a multigenerational exposure model to evaluate the effects of maternal wood-smoke-derived PM_2.5_ exposure on fetal intramembranous ossification in Sprague–Dawley rats (Figure 8). Generation 1 (G1) females were obtained from pregnant founder dams housed in either filtered air (FA) or non-filtered air (NFA) chambers during gestation. After birth, G1 females remained under their assigned environmental condition until reproductive maturity. Twelve healthy nulliparous G1 females exhibiting regular 4–5 day estrous cycles were selected for breeding and paired with sexually mature non-exposed males. The resulting Generation 2 (G2) female offspring remained under the environmental condition corresponding to their maternal exposure lineage until reproductive maturity. Twelve healthy nulliparous G2 females exhibiting regular estrous cyclicity were then selected for the experimental pregnancy protocol. Selected G2 females were paired overnight (18:00–08:00 h) with sexually mature non-exposed males at a 1:1 male:female ratio. Gestational day 0 (GD0) was defined by the detection of vaginal copulation plugs or spermatozoa in vaginal smears during morning examination. At GD0, pregnant G2 females were allocated to either FA or NFA gestational exposure according to their pregestational exposure history, generating a 2 × 2 factorial design. This allocation generated four experimental groups: FA/FA, FA/NFA, NFA/FA, and NFA/NFA. The first term indicates the pregestational exposure lineage of the G2 female, whereas the second term indicates the gestational exposure condition during G3 fetal development. This design allowed assessment of the independent and combined effects of pregestational exposure across the maternal lineage and gestational exposure during G3 fetal development on fetal skeletal formation [10,11]. Selection of females with regular 4–5 day estrous cycles was based on established criteria in reproductive toxicology, in which cycle regularity serves as a functional indicator of hypothalamic–pituitary–ovarian axis integrity. Females exhibiting irregular cycles were excluded because estrous irregularity is associated with altered ovarian steroidogenesis and reduced implantation rates, which constitute confounding variables in studies of prenatal development [11]. Non-exposed adult males were used exclusively as breeding partners; the potential contribution of paternal PM_2.5_ exposure to fetal skeletal outcomes is acknowledged as an avenue for future investigation.

#### 4.4.2. Experimental Groups

Pregnant G2 females (*n* = 24 total) were allocated into four experimental groups (*n* = 6 pregnant females per group) according to their pregestational exposure lineage and gestational exposure condition:

- FA/FA (Control): G2 females derived from the filtered-air lineage and gestated under filtered air.
- FA/NFA: G2 females derived from the filtered-air lineage and gestated under non-filtered air.
- NFA/FA: G2 females derived from the non-filtered-air lineage and gestated under filtered air.
- NFA/NFA: G2 females derived from the non-filtered-air lineage and gestated under non-filtered air.

This 2 × 2 exposure structure was designed to compare fetal outcomes across combinations of pregestational exposure lineage and gestational exposure condition. The pregnant dam/litter was recognized as the primary experimental unit in reproductive toxicology, because fetuses from the same mother share maternal physiology, placental environment, genetic background, and intrauterine conditions and therefore cannot be considered fully independent biological replicates [46,47]. Individual G3 fetuses were considered fetal-level observational units. For fetal morphometric and immunohistochemical endpoints, one fetus per litter was selected for each experimental group (*n* = 6 L per group; six fetuses per group, each from an independent litter), ensuring that fetal-level observations were statistically independent and that intra-litter correlation did not affect these analyses. Sample sizes differed by assay due to methodological constraints: morphometric and immunohistochemical outcomes were evaluated in six G3 fetuses per group, each from an independent litter (n=6 L per group; one fetus per litter); quantitative micro-CT BMD analysis was restricted to the FA/FA and NFA/NFA groups, with *n* = 5 fetuses per group, each from an independent litter; and whole-mount skeletal staining was performed on five fetuses per group. The potential influence of intra-litter dependence is acknowledged as a methodological limitation of the statistical approach.

#### 4.4.3. Sample Size Determination

Sample size estimation was performed a priori using G*Power software (version 3.1.9.7; Heinrich Heine University, Düsseldorf, Germany), assuming two-tailed hypothesis testing, a statistical significance threshold of α = 0.05, statistical power ≥ 80% (β = 0.20), and an expected large effect size based on preliminary pilot data. Although the initial calculation indicated that a larger number of fetal-level observations would be required to detect moderate-to-large differences with adequate statistical power, the final sample size was constrained by the number of available pregnant G2 females and fetuses meeting the inclusion criteria. Therefore, the study was interpreted as an experimental analysis with limited fetal-level sample size, and the pregnant dam/litter was considered the primary biological unit for interpretation. Throughout the experimental period, all pregnant females completed the protocol without exclusions. No maternal deaths, pregnancy losses or premature deliveries were recorded. Animals maintained normal health status, as assessed by daily observation of general activity, food and water consumption, and body weight progression.

#### 4.4.4. Animal Housing and Husbandry

Throughout the experimental period, animals were maintained under standardized environmental conditions with ad libitum access to pelleted rodent chow (Prolab RMH 3000; LabDiet, St. Louis, MO, USA) and filtered municipal water delivered through automatic watering systems. Cages (polycarbonate; 48 cm × 27 cm × 20 cm) housed 2–3 animals per cage with corn-cob bedding (renewed twice weekly) and environmental enrichment (PVC tubes, wooden chew blocks). Room temperature (20–25 °C), relative humidity (40–60%), and photoperiod (12:12 h light/dark; lights on 07:00 h) were continuously monitored and maintained within specified ranges. Animal health status was assessed daily by trained personnel; veterinary consultation was available for any animals displaying signs of illness or distress.

### 4.5. Fetal Collection and Morphometric Analysis

#### 4.5.1. Cesarean Section Protocol

At gestational day 21 (GD21), pregnant G2 females were euthanized following OECD Test Guideline 414 protocols for prenatal developmental toxicity studies [48]. Cesarean section was performed immediately under aseptic conditions in a temperature-controlled surgical area (22–24 °C). The uterus was exteriorized through midline laparotomy, and each fetal position within uterine horns was recorded. Fetuses were carefully extracted with intact placental-umbilical attachments, immediately separated from extraembryonic membranes and gently dried on sterile gauze. Pregnant G2 females were euthanized by deep isoflurane inhalation anesthesia (induction: 5% isoflurane in oxygen; maintenance: 2–3% isoflurane via nose cone) followed by cervical dislocation as a confirmatory physical method, in accordance with the recommendations of the Guide for the Care and Use of Laboratory Animals (National Research Council, 8th edition, 2011) and the AVMA Guidelines for the Euthanasia of Animals (2020 edition; [49]). This combined protocol (chemical induction + physical confirmation) is consistent with AVMA-approved methods [49] for rodents and ensures rapid loss of consciousness with minimal distress prior to tissue collection.

#### 4.5.2. Fetal Morphometry

Morphometric assessment was conducted on fresh specimens within 15 min post-extraction. Each fetus was weighed (analytical balance: precision ±0.001 g; Sartorius BP211D, Göttingen, Germany) and measured for crown–rump length (CRL). For CRL, fetuses were positioned in left lateral decubitus with vertebral column in neutral alignment and limbs extended. CRL was measured from cranial vertex to sacral prominence using a digital caliper (resolution 0.01 mm; Mitutoyo CD-6”CSX, Kawasaki, Japan) with rounded faces to prevent soft tissue compression. Two blinded observers performed three consecutive measurements per fetus; the arithmetic mean served as the representative value. Measurement precision was assessed by calculating intraobserver and interobserver coefficients of variation (CVs) in a subset of fetuses randomly selected from the total sample. Acceptance criteria were defined as CV <5% between repeated measurements and <3% deviation from the reference measurement obtained under stereomicroscopic validation (Mitutoyo CD-6”CSX, Kawasaki, Japan; 2×; NIST-traceable stage micrometer). Observers showing systematic bias >2% underwent retraining. All procedures were performed under complete blinding using dual-coded specimen labels. Group allocation keys remained secured until completion of all measurements and statistical analyses.

### 4.6. Skeletal Imaging and Analysis

The skeletal analysis was primarily focused on cranial bones formed predominantly by intramembranous ossification, including the frontal, parietal, interparietal, and occipital regions of the fetal neurocranium. Therefore, radiographic, micro-CT, whole-mount staining, histological, and immunohistochemical assessments were interpreted in relation to membranous neurocranial ossification. Endochondral skeletal elements were not considered primary outcomes and were interpreted only when explicitly evaluated.

#### 4.6.1. Whole-Mount Skeletal Preparation

Double-staining with alcian blue (cartilage) and alizarin red S (bone) [50] provided direct visualization of mineralization extent, complementary to tomographic methods [51]. Five fetuses per group were eviscerated, washed 12 h in distilled water, then cleared in 5% KOH (5 days, 20–22 °C). This alkaline hydrolysis digested non-mineralized tissues while preserving skeletal and cartilaginous structures. Specimens were stained in 0.1% alizarin red S (6 h). Alizarin red S binds stoichiometrically to calcium in hydroxyapatite, producing coloration proportional to mineralization. After serial washes (24–48 h), specimens underwent glycerol gradient dehydration (25%, 50%, 75%, 100%) and storage in pure glycerol in sealed, light-protected vials. Examination used a Leica EZ4 stereomicroscope (Leica Microsystems, Heerbrugg, Switzerland) with transmitted/reflected illumination. Digital photomicrographs were acquired at standardized magnifications. Cranial flat bones were assessed for: (1) bone margin sharpness; (2) staining uniformity (inner/outer tables); (3) suture morphology (coronal, sagittal, lambdoid); (4) calvarial bone relationships (frontal, parietal, interparietal, occipital); (5) facial bone ossification (maxilla, nasal); and (6) hypomineralization patterns. Staining patterns were compared against established atlases for GD21-22 rat fetal skeletal development [51]. Intense red = mineralized bone; unstained = cartilaginous templates; intermediate = active mineralization fronts.

#### 4.6.2. Digital Radiographic Assessment

Whole-body fetal radiography was performed using a high-frequency digital X-ray system (JADE, DRGEM, Wonju, Republic of Korea; 4 kW, 100 kHz). Exposure parameters were standardized as follows: 41 kV, 1.0 mAs, focal-spot-to-detector distance 98 cm, and exposure duration adjusted according to specimen size. Images were acquired in ventrodorsal and lateral projections under consistent geometric conditions to minimize magnification-related variation. Evaluation criteria: (1) global skeletal architecture and symmetry; (2) calvarial mineralization degree; (3) secondary ossification centers; (4) bone margin sharpness; (5) bilateral symmetry; (6) cranial suture delineation; and (7) bone mineral density homogeneity. Findings were compared against published fetal skeletal atlases for gestational day 21 rats [51].

#### 4.6.3. Micro-Computed Tomography (Micro-CT)

High-resolution 3D characterization was performed using micro-CT [51,52]. Acquisition parameters: SkyScan 1278 (Bruker microCT, Kontich, Belgium; v1.0.5) operated at 59 kV, 692 μA with 0.5 mm aluminum filter. Isotropic voxel size: 51.489 μm. Exposure time: 187 ms per projection. Total rotation: 360°; angular step: 0.4°; number of projections: 900. Frame averaging: 3 frames per projection. Ring artifact correction: level 10. Beam hardening correction: 40%. Scan duration: approximately 1 h 35 min per specimen. Raw projection data were reconstructed into axial cross-sections (16-bit TIFF format) using NRecon software (v1.7.4.6, Bruker, Kontich, Belgium) with the following settings: smoothing kernel 2, ring artifact reduction 10, beam hardening correction 40%, and misalignment compensation enabled. Reconstructed slices were converted to NRRD format and imported into 3D Slicer (v5.6.2; http://www.slicer.org) [53,54] for visualization and semi-automated segmentation using the SlicerMorph extension. The selected voxel size (51.489 μm) provided adequate spatial resolution for regional volumetric assessment of fetal neurocranial bones at GD21, where bone thickness typically ranges from 150 to 400 μm [51]. However, this resolution was insufficient to resolve fine cortical microarchitecture or trabecular detail. Partial volume effects at bone edges, sutures, and ossification fronts were expected and mitigated by restricting quantitative ROI placement to homogeneous mineralized regions within bone centers. BMD estimates derived at this resolution should be interpreted as regional volumetric measures rather than cortical or trabecular microstructural parameters. Micro-CT-derived outcomes included qualitative assessment of ossification continuity, regional mineralization patterns, bone surface continuity, bone–soft tissue boundaries, ossification fronts, cranial suture definition, cranial vault alignment, and hypomineralized regions, as well as quantitative volumetric bone mineral density (BMD) estimation in selected neurocranial bones. Anatomical nomenclature followed standard terminology for GD21 *Rattus norvegicus* skeletal development [51]. Volumetric BMD was quantified using CTAn software (Bruker v.1.18; Bruker microCT, Kontich, Belgium)) following standardized protocols [55]. Cylindrical regions of interest (ROIs; 2 mm diameter, 0.5 mm height) were manually positioned at the medial-central region of frontal, parietal, interparietal, and occipital bones. When required by bone size or shape, ROI placement was adjusted while preserving the same anatomical location and avoiding sutural or marginal regions. ROI placement criteria ensured complete containment within the mineralized bone area, verified by visual inspection in coronal, sagittal, and transverse planes; avoidance of bone edges, sutures, and non-mineralized regions; and anatomical consistency across specimens. For each bone, five spatially distinct ROI measurements were acquired per fetus and treated as technical replicates. Each fetus contributed a single BMD value per bone, calculated as the mean of five ROI measurements, thereby avoiding ROI-level pseudoreplication.

BMD calibration was performed using hydroxyapatite (HA) phantoms (250 and 750 mg HA/cm^3^) scanned under identical acquisition parameters. Threshold segmentation employed a global threshold of 60–255 grayscale values, validated by visual inspection of bone–soft tissue boundaries. BMD was expressed as mg HA/cm^3^. Quantitative micro-CT BMD analysis was restricted to the FA/FA (control) and NFA/NFA (continuous-exposure) groups, with n=5 fetuses per group, each obtained from an independent litter. This maximal-contrast design was selected to compare the minimal and maximal cumulative PM_2.5_ exposure conditions and to maximize statistical power for detecting mineralization deficits. Because each fetus originated from a distinct litter, intra-litter dependence did not affect this comparison. This analytical decision was based on three justifications. FA/FA and NFA/NFA represent exposure spectrum extremes, corresponding to minimal versus maximal cumulative PM_2.5_ exposure [56,57], maximizing biological contrast and statistical power to detect mineralization deficits. Qualitative assessment of all four groups identified the interparietal and occipital bones as the most vulnerable neurocranial sites; concentrating the quantitative BMD analysis on maximal-contrast groups enhanced detection sensitivity for region-specific deficits [55,58]. Micro-CT scanning and quantitative BMD analysis are time- and resource-intensive, and restricting quantitative analysis to the two groups most likely to differ allowed efficient allocation of analytical resources. Because FA/NFA and NFA/FA groups were excluded from quantitative micro-CT analysis, BMD results cannot be used to estimate independent pregestational effects, gestational effects, or pregestational × gestational interaction. The FA/FA versus NFA/NFA comparison provides evidence of mineralization deficits under continuous PM_2.5_ exposure but does not permit formal factorial decomposition of exposure timing effects. Such decomposition would require inclusion of all four experimental groups in the quantitative analysis.

Radiography, micro-CT and alizarin staining provided complementary assessment: radiography for rapid screening; micro-CT for 3D quantification; alizarin for direct mineralization confirmation [51,52].

### 4.7. Histological Analysis

#### 4.7.1. Histological Evaluation

Parietal bone was selected for the histological analysis as a representative intramembranous bone of the neurocranium, offering consistent anatomical orientation, reproducible sectioning plane, and accessibility for serial sampling. Samples were fixed in 10% neutral buffered formalin, decalcified in 10% EDTA solution (pH 7.4) for 7–10 days with weekly solution changes, dehydrated through graded ethanol series, cleared in xylene, and embedded in paraffin. Serial coronal sections (5 μm thickness) were obtained from the central region of the parietal bone using a rotary microtome. Three non-consecutive sections per fetus, spaced 50 μm apart, were mounted on glass slides and stained with hematoxylin and eosin (H&E) following standard protocols for bone tissue visualization. For each section, three microscopic fields were systematically evaluated at anatomically homologous regions corresponding to the central trabecular zone, avoiding bone edges and suture regions. Qualitative histological assessment focused on structural and cellular features indicative of intramembranous ossification maturity. Immature bone tissue (*textus osseus immaturus*) was evaluated for: (1) trabecular organization and structural margins; (2) matrix tinctorial properties reflecting osteoid–bone matrix organization; (3) osteoid (*osteoidum*) distribution and abundance; (4) osteocyte (*osteocyti*) incorporation into bone lacunae and spatial distribution; (5) osteoprogenitor cell (*cellulae osteogenicae*) presence and localization at the trabecular periphery; and (6) osteoblast (*osteoblasti*) density and distribution pattern along developing bone surfaces. Structural maturity was descriptively classified according to matrix homogeneity, trabecular continuity, osteoid organization, and cellular differentiation patterns consistent with progressive or delayed ossification. Images were acquired using a Leica DM750 microscope equipped with ICC50W camera (5 MP) and LAS software (v3.4.0, Leica Microsystems, Heerbrugg, Switzerland) at 100×, 200×, and 400× magnifications for documentation and comparative qualitative analysis across experimental groups.

#### 4.7.2. Immunohistochemistry

Immunodetection was performed on 6 μm paraffin sections from the parietal bone (n=5 fetuses per group, each from independent litters). Sections were deparaffinized, rehydrated and subjected to heat-induced epitope retrieval in 10 mM citrate buffer (pH 6.0) at 95 °C for 20 min. Endogenous peroxidase activity was blocked with 3% H_2_O_2_ for 10 min, followed by blocking of non-specific binding with 5% normal goat serum in PBS for 30 min at room temperature. Primary antibodies diluted in 1% BSA/PBS were incubated overnight at 4 °C in humidified chambers: HIF-1α (1:50, mouse monoclonal, clone H1alpha67, cat# sc-13515, Santa Cruz Biotechnology, Dallas, TX, USA; validated for rat), BMP-2 (1:100, rabbit polyclonal, cat# NBP1-19751, Novus Biologicals, Centennial, CO, USA; validated for rat), TGF-β1 (1:200, mouse monoclonal, clone TB21, cat# NB600-1432, Novus Biologicals; validated for rat), FGF-R1 (1:100, mouse monoclonal, clone 4FR8A, cat# NBP2-67415, Novus Biologicals; validated for rat), and type I collagen (COL-1, 1:100, rabbit polyclonal, cat# NB600-408, Novus Biologicals; validated for rat). Negative controls (primary antibody omitted) and positive controls (adult rat bone) were processed in parallel. Detection was performed using an HRP/DAB system (IHC Select, DAB150, Merck, Darmstadt, Germany) with 5 min chromogen development time. Sections were counterstained with hematoxylin, dehydrated, mounted in Entellan (Merck, Darmstadt, Germany) and dried at 37 °C. Immunoreactivity was quantified from three non-consecutive sections per fetus, spaced 50 μm apart. For each section, three microscopic fields at 200× magnification were captured from anatomically homologous regions corresponding to the central trabecular zone. Digital images were analyzed using ImageJ software (v1.54f; National Institutes of Health, Bethesda, MD, USA). DAB signal was isolated by color deconvolution and a fixed threshold (validated against negative controls) was applied to all images. For HIF-1α, nuclear and cytoplasmic compartments were analyzed separately to distinguish transcriptionally active (nuclear) from inactive (cytoplasmic) signal. For COL-1, an immunoreactive area was interpreted as extracellular matrix deposition rather than cellular expression. Results were expressed as immunoreactive area fraction (%) [9,59]. Each fetus contributed a single value per marker (mean of nine fields: three sections × three fields per section). Two blinded observers independently analyzed all images; inter-rater reproducibility was validated using the intraclass correlation coefficient (two-way random-effects model, absolute agreement; ICC > 0.85).

### 4.8. Statistical Analysis

Data were analyzed in R version 4.5.1 (R Foundation for Statistical Computing, Vienna, Austria) and GraphPad Prism (version 10.0; GraphPad Software, San Diego, CA, USA), as appropriate. Descriptive statistics are expressed as mean ± SEM, unless otherwise indicated. Normality was assessed using the D’Agostino–Pearson test. Homogeneity of variance was evaluated by visual inspection of residual plots and, when applicable, Levene’s test. No outliers were excluded. Statistical analyses were performed using coded group identifiers, and group allocation keys remained secured until completion of the primary analyses. For fetal morphometric and immunohistochemical parameters, a two-way factorial ANOVA was used to evaluate the effects of pregestational exposure, gestational exposure, and their interaction. The two fixed factors were pregestational exposure (FA vs. NFA) and gestational exposure (FA vs. NFA), generating the four experimental groups FA/FA, FA/NFA, NFA/FA, and NFA/NFA. Type III sums of squares were used to evaluate main and interaction effects in the presence of potential group imbalance [60]. Estimated marginal means (EMMs) were computed using the emmeans package [61], and pairwise comparisons among the four experimental groups were performed using Tukey’s HSD adjustment. For quantitative micro-CT bone mineral density (BMD) analysis, comparisons were restricted to the FA/FA control group and the NFA/NFA continuous-exposure group, as described above. Because FA/NFA and NFA/FA groups were not included in the quantitative micro-CT analysis, BMD results were interpreted as targeted comparisons between minimal and maximal cumulative ${\mathrm{PM}}_{2.5}$ exposure conditions and were not used to estimate independent pregestational effects, gestational effects, or pregestational × gestational interaction effects. ROI-level measurements were treated as technical replicates and averaged to obtain one fetal-level BMD value per bone before statistical analysis. Between-group comparisons were performed using unpaired *t*-tests when assumptions of normality and homogeneity of variance were met. Interaction effects were visualized using interaction plots showing mean ± SEM by gestational exposure, stratified by pregestational exposure. Individual distributions were displayed as boxplots with jittered data points. R analyses used the car package for Type III ANOVA [62], emmeans for estimated marginal means [61], ggplot2 for graphical visualization [63], and dplyr/tidyr for data handling. The factorial structure was retained because it allows the evaluation of whether pregestational exposure modifies the effect of gestational exposure, a biologically relevant question in developmental programming frameworks [64,65]. However, the pregnant dam/litter is the primary biological unit in reproductive toxicology, because fetuses from the same litter share maternal physiology, genetic background, placental environment, and intrauterine conditions and therefore cannot be considered fully independent biological replicates [46,47]. For fetal morphometric and immunohistochemical endpoints, however, one fetus per litter was included in each experimental group (n=6 L per group). Thus, each observation was statistically independent, and intra-litter dependence did not apply to these analyses; factorial ANOVA without a litter random effect was therefore the appropriate model. For quantitative micro-CT BMD, the FA/FA versus NFA/NFA comparison (n=5 fetuses per group, each from an independent litter) was performed using an unpaired *t*-test, consistent with Table 2. Because all analyzed endpoints used one fetus per litter, the litter and the analyzed fetus represented the same statistical unit in this dataset, and no nested or mixed-effects modeling was required. Future studies incorporating multiple fetuses per litter should employ linear mixed-effects models or generalized estimating equations to explicitly account for the nested fetal structure [47,66,67,68].

## 5. Conclusions

Conclusions regarding PM_2.5_-associated skeletal effects in this study are restricted to fetal neurocranial intramembranous ossification. Endochondral skeletal elements were not systematically quantified and therefore any reference to generalized skeletal toxicity or broad skeletal development has been removed from the revised text. Maternal exposure to wood-smoke-derived PM_2.5_ was associated with delayed fetal neurocranial intramembranous ossification in Sprague–Dawley rats, particularly under continuous pregestational and gestational exposure. This phenotype included reduced fetal growth, altered craniofacial mineralization, lower regional mineral density, delayed histological maturation, increased HIF-1α immunoreactivity, exposure-window-dependent modulation of TGF-β, and reduced COL-I immunoreactivity. The posterior neurocranium, especially the occipital and interparietal bones, appeared particularly sensitive to this exposure pattern, supporting its value as a morphological readout of environmentally induced developmental disruption. These findings support the hypothesis that biomass-derived PM_2.5_ may interfere with fetal skeletal maturation by altering the relationship between extracellular matrix organization and mineralization. However, because oxygen tension, oxidative stress, vascularization, Smad signaling, collagen gene expression, and osteoblast functional activity were not directly measured, the proposed PM_2.5_–placenta–hypoxia-associated signaling–ossification axis remains an inferential mechanistic model requiring further validation. Overall, this study identifies fetal neurocranial intramembranous ossification as a previously underexplored target of maternal air-pollution exposure and highlights the pregestational and gestational periods as relevant windows of skeletal developmental vulnerability.

## Figures and Tables

**Table 1:** Selected evidence on ${\mathrm{PM}}_{2.5}$ exposure and fetal/postnatal skeletal development.

Study	Species	Bone Tissue	Exposure Window	Main Finding
Ge et al. [16]	Mouse	Femur (endochondral)	Gestational/adult	↓ Femoral length, BMD
Cao et al. [17]	Human	Femur (endochondral)	Gestational	↓ Fetal length (ultrasound)
Sram et al. [18]	Human	General skeleton	Postnatal	↓ Growth (0–3 years)

Selected representative studies are shown to illustrate the predominance of endochondral or general skeletal outcomes in the available literature. Note: ↓ indicates a decrease. Abbreviation: BMD, bone mineral density.

**Table 2:** Quantitative micro-CT analysis of bone mineral density in fetal neurocranial bones at 21 days post-fertilization.

Bone Region	FA/FA	NFA/NFA	Reduction vs. FA/FA (%)	*p*-Value
Frontal	641±49	618±56	3.6	0.42
Parietal	635±52	609±61	4.1	0.38
Interparietal	612±51 ^a^	485±63 ^b^	20.8	0.0086 *
Occipital	628±58 ^a^	501±71 ^b^	20.2	0.0123 *

Bone mineral density (BMD) values are expressed as mean ± standard deviation in mg hydroxyapatite (HA)/cm^3^. Five ROI measurements per fetus were averaged to yield one BMD value per fetus per bone (technical replicates; not used as independent observations). Statistical comparisons were performed on fetal-level means. Biological *n*: FA/FA =5 fetuses; NFA/NFA =5 fetuses (n=25 ROI measurements per group represent technical replicates only and are reported for transparency). Reduction values indicate the percentage decrease relative to the FA/FA control group. Different superscripts indicate statistically significant differences between groups within the same bone region (unpaired *t*-test, α=0.05). * indicates p<0.05. FA/FA: pregestational and gestational filtered air; NFA/NFA: pregestational and gestational non-filtered air. Analysis was performed using CTAn software (v.1.18, Bruker, Kontich, Belgium).

**Table 3:** Two-way factorial ANOVA for immunohistochemical markers in fetal parietal bone.

Marker	Main Effects and Interaction			Estimated Marginal Means (% Area)			
	Preg.	Gest.	P×G	FA/FA	FA/NFA	NFA/FA	NFA/NFA
HIF-1α	0.001	0.030	0.334	21.99±4.36	33.81±2.94	38.79±2.18	56.86±2.94
COL1	0.151	0.032	0.003	38.42±2.85	30.25±2.32	44.67±3.18	19.05±2.50
TGF-β	0.624	0.502	0.049	23.26±2.94	26.38±3.56	19.89±6.17	41.43±4.88
BMP2	0.231	0.157	0.082	23.68±2.26	18.14±3.09	19.33±2.77	24.10±3.30
FGF-R1	0.213	0.634	0.932	35.79±3.59	33.05±4.40	28.87±4.08	25.48±3.11

Type III two-way ANOVA. Preg.: pregestational exposure; Gest.: gestational exposure; P×G: interaction between pregestational and gestational exposures. FA/FA: control; FA/NFA: gestational exposure; NFA/FA: pregestational exposure; NFA/NFA: dual exposure. HIF-1α: hypoxia-inducible factor 1-alpha; COL1: type I collagen; TGF-β: transforming growth factor beta; BMP2: bone morphogenetic protein 2; FGF-R1: fibroblast growth factor receptor 1. Values represent estimated marginal means ± standard error of the mean (SEM).

## Data Availability

The original contributions presented in this study are included in the Supplementary Materials. Further inquiries can be directed to the corresponding author.

## Supplementary Materials

The following supporting information can be downloaded at: https://www.mdpi.com/article/10.3390/ijms27135715/s1.

## Abbreviations

The following abbreviations are used in this manuscript:

ARRIVE	Animal Research: Reporting of In Vivo Experiments
BMD	Bone mineral density
BV/TV	Bone volume fraction
CO	Carbon monoxide
CRL	Crown–rump length
DOHaD	Developmental origins of health and disease
FA	Filtered air
GD	Gestational day
HEPA	High-efficiency particulate air
HIF-1α	Hypoxia-inducible factor 1-alpha
IHC	Immunohistochemistry
Micro-CT	Micro-computed tomography
NFA	Non-filtered air
${\mathrm{NO}}_{2}$	Nitrogen dioxide
PM	Particulate matter
${\mathrm{PM}}_{2.5}$	Fine particulate matter with an aerodynamic diameter ≤2.5 μm
${\mathrm{PM}}_{10}$	Particulate matter with an aerodynamic diameter ≤10 μm
SD	Sprague–Dawley
SHH	Sonic hedgehog
SPF	Specific pathogen-free
VOI	Volume of interest
WNT	Wingless/integrated signaling pathway

## References

[1] Cao Z.J., Zhao Y., Wang S.M., Zhang D.L., Zhou Y.C., Liu W.N., Yang Y.Y., Hua J. (2021). Prenatal exposure to fine particulate matter and fetal growth: A cohort study from a velocity perspective. Chemosphere.

[2] Huang Z., Wu J., Qiu Y., Lin J., Huang W., Ma X., Zhang H., Yang X. (2023). Association between gestational exposure and risk of orofacial clefts: A systematic review and meta-analysis. BMC Pregnancy Childbirth.

[3] Bové H., Bongaerts E., Slenders E., Bijnens E.M., Saenen N.D., Gyselaers W., Van Eyken P., Plusquin M., Roeffaers M.B.J., Ameloot M. (2019). Ambient black carbon particles reach the fetal side of human placenta. Nat. Commun..

[4] Tian Y., Hu Y., Hou X., Tian F. (2024). Impacts and mechanisms of PM2.5 on bone. Rev. Environ. Health.

[5] Torkashvand J., Jonidi Jafari A., Pasalari H., Shahsavani A., Oshidari Y., Amoohadi V., Kermani M. (2022). The potential osteoporosis due to exposure to particulate matter in ambient air: Mechanisms and preventive methods. J. Air Waste Manag. Assoc..

[6] Long F., Ornitz D.M. (2013). Development of the endochondral skeleton. Cold Spring Harb. Perspect. Biol..

[7] Ornitz D.M., Marie P.J. (2015). Fibroblast growth factor signaling in skeletal development and disease. Genes Dev..

[8] Yang D.C., Yang M.H., Tsai C.C., Huang T.F., Chen Y.H., Hung S.C. (2011). Hypoxia inhibits osteogenesis in human mesenchymal stem cells through direct regulation of RUNX2 by TWIST. PLoS ONE.

[9] Villarroel F., Ponce N., Gómez F.A., Muñoz C., Ramírez E., Nualart F., Salinas P. (2024). Exposure to fine particulate matter 2.5 from wood combustion smoke causes vascular changes in placenta and reduce fetal size. Reprod. Toxicol..

[10] Villarroel F., Ramírez E., Ponce N., Nualart F., Salinas P. (2025). Impact of PM2.5 Emitted by Wood Smoke on the Expression of Glucose Transporter 1 (GLUT1) and Sodium-Dependent Vitamin C Transporter 2 (SVCT2) in the Rat Placenta: A Pregestational and Gestational Exposure Study. Antioxidants.

[11] Salinas P., Ponce N., Del Sol M., Vásquez B. (2025). Impact of PM2.5 Exposure from Wood Combustion on Reproductive Health: Implications for Fertility, Ovarian Function, and Fetal Development. Toxics.

[12] Tao S., Zhang X., Tian F., Pan B., Peng R., Wang Y., Xia M., Yang M., Hu J., Kan H. (2022). Maternal exposure to ambient PM2.5 causes fetal growth restriction via the inhibition of spiral artery remodeling in mice. Ecotoxicol. Environ. Saf..

[13] Wu H., Kioumourtzoglou M.A., Just A.C., Kloog I., Sanders A., Svensson K., McRae N., Tamayo-Ortiz M., Solano-González M., Wright R.O. (2020). Association of ambient PM2.5 exposure with maternal bone strength in pregnant women from Mexico City: A longitudinal cohort study. Lancet Planet. Health.

[14] Du J., Cui H., Zhao Y., Xue H., Chen J. (2024). Exposure to air pollution might decrease bone mineral density and increase the prevalence of osteoporosis: A Mendelian randomization study. Osteoporos. Int..

[15] Grzonkowska M., Baumgart M., Badura M., Wiśniewski M., Szpinda M. (2021). Quantitative anatomy of the fused ossification center of the occipital squama in the human fetus. PLoS ONE.

[16] Ge Q., Yang S., Qian Y., Chen J., Yuan W., Li S., Wang P., Li R., Zhang L., Chen G. (2023). Ambient PM2.5 Exposure and Bone Homeostasis: Analysis of UK Biobank Data and Experimental Studies in Mice and in Vitro. Environ. Health Perspect..

[17] Cao Z., Meng L., Zhao Y., Liu C., Yang Y., Su X., Fu Q., Wang D., Hua J. (2019). Maternal exposure to ambient fine particulate matter and fetal growth in Shanghai, China. Environm. Health.

[18] Sram R.J., Binkova B., Dostal M., Merkerova-Dostalova M., Libalova H., Milcova A., Rossner P., Rossnerova A., Schmuczerova J., Svecova V. (2013). Health impact of air pollution to children. Int. J. Hyg. Environ. Health.

[19] Gao J., Luo M., Zhao S., Wang H., Li X., Xu P., Ma W., Liu C. (2022). Effect of PM2.5 exposure on gestational hypertension, fetal size in preeclampsia-like rats. Environ. Sci. Pollut. Res. Int..

[20] Hussein A.I., Carroll D., Bui M., Wolff A., Matheny H., Hogue B., Lybrand K., Cooke M., Bragdon B., Morgan E. (2023). Oxidative metabolism is impaired by phosphate deficiency during fracture healing and is mechanistically related to BMP induced chondrocyte differentiation. Bone Rep..

[21] Scheepers L.E., Binter A.C., Santos S., Petricola S., Rivadeneira F., Jaddoe V.W., Guxens M., Johnston F.H. (2025). Air pollution and bone health outcomes: Periods of susceptibility from pregnancy to childhood. Environ. Int..

[22] Lapehn S., Paquette A.G. (2022). The Placental Epigenome as a Molecular Link Between Prenatal Exposures and Fetal Health Outcomes Through the DOHaD Hypothesis. Curr. Environ. Health Rep..

[23] Dallas S.L., Bonewald L.F. (2010). Dynamics of the transition from osteoblast to osteocyte. Ann. N. Y. Acad. Sci..

[24] da Silva A.F., Lima F.J., Moreira A.R., Silva C.d.N., de Oliveira I.B., Callera A.F., Porfirio A.L., Alves L.H.V., Tibério I.d.F.L.C., Velosa A.P.P. (2025). Cigarette Smoke Exposure Leads to Organic and Mineral Bone Component Changes: The Importance of Rho Kinase Function in These Events. Cells.

[25] Qiao J., Liu A., Sun C., Liu Q. (2025). HIF1A overexpression promotes osteoblast differentiation through activation of autophagy to alleviate osteoporosis. Sci. Rep..

[26] Chen Q., Wang B., Liang H., Xu H., Zhang K., Hao Y. (2026). Bilobalide attenuates steroid-induced osteonecrosis of the femoral head by upregulating the ERK/HIF-1*α* signaling pathway and promoting angiogenesis-osteogenesis coupling. Sci. Rep..

[27] Lang J., Morya V.K., Kwak M.K., Park S.H., Noh K.C. (2025). Molecular crosstalk in SP7-mediated osteogenesis: Regulatory mechanisms and therapeutic potential. Osteoporos. Sarcopenia.

[28] Yang J., Han C., Ye J., Hu X., Wang R., Shen J., Li L., Hu G., Shi X., Jia Z. (2024). PM_2.5_ exposure inhibits osteoblast differentiation by increasing the ubiquitination and degradation of Smad4. Toxicol. Lett..

[29] Hu X., Zhao Y., He T., Gao Z.X., Zhang P., Fang Y., Ge M., Xu Y.Q., Pan H.F., Wang P. (2023). Causal Relationships between Air Pollutant Exposure and Bone Mineral Density and the Risk of Bone Fractures: Evidence from a Two-Stage Mendelian Randomization Analysis. Toxics.

[30] Nicholls L.A., Zeile K.A., Scotto L.D., Ryznar R.J. (2025). Timing of dietary effects on the epigenome and their potential protective effects against toxins. Epigenetics.

[31] Obrycka P., Soczyńska J., Butyńska K., Frątczak A., Hałaburdo J., Gawełczyk W., Woźniak S. (2026). Impact of Early-Life Environmental Exposures and Potential Transgenerational Influence on the Risk of Coronary Artery Disease and Heart Failure. Cells.

[32] Polverino F., Sin D.D. (2025). The Developmental Origins of Asthma and COPD. Annu. Rev. Physiol..

[33] Lee K.K., Changoor A., Grynpas M.D., Mitchell J. (2026). Increased osteoblast G*α*_11_ level compromises bone healing quality by suppressing high-density bone formation. Bone.

[34] Chen Y., Shen H., Wang Z., Zhu W., Lu W., Shen J., Li W., Zhang Y., Yang G., Wu Y. (2026). Mechanistic insights into osteotoxicity induced by early-life lead exposure: Evidence from metabolomics and network toxicology. Ecotoxicol. Environ. Saf..

[35] Kang N., Yang Z., Petrick L.M., Rahman M.M., Pavlovic N., Lurmann F.W., Martinez M.P., Yu X., Chow T., Eckel S.P. (2026). Newborn metabolomics linking prenatal air pollution exposure and autism spectrum disorder risk in children. J. Expo. Sci. Environ. Epidemiol..

[36] Aik J., Lau H.X., Woo M., Shek L.P.C., Lee B.W., Goh A.E.N., Tan K.H., Yap F.K.P., Gluckman P., Chong Y.S. (2026). Meteorological, ozone, maternal and individual-level risk factors for childhood diseases in Singapore: A prospective birth cohort study from 2009 to 2019. Ecotoxicol. Environ. Saf..

[37] Padula A.M., Mayo J.A., Lurmann F.W., Pavlovic N.R., Shaw G.M. (2026). Wildland fire smoke and birth defects in California. J. Expo. Sci. Environ. Epidemiol..

[38] Kim E.J., Bae J.G., Koo E.J. (2026). Prenatal Fine Particulate Matter (PM2.5) Exposure and the Risk of Pediatric Inguinal Hernia or Hydrocele: A Retrospective Cohort Study. J. Clin. Med..

[39] IQAir (2021). 2021 World Air Quality Report: Region and City PM2.5 Ranking.

[40] Fuchs L.F.P., Veras M.M., Saldiva P.H.N., Sasso G.R.d.S., Carvalho K.C., Simões M.d.J., Soares J.M., Baracat E.C. (2017). Ambient levels of concentrated PM2.5 affects cell kinetics in adrenal glands: An experimental study in mice. Gynecol. Endocrinol..

[41] Yan Y.H., C-K Chou C., Wang J.S., Tung C.L., Li Y.R., Lo K., Cheng T.J. (2014). Subchronic effects of inhaled ambient particulate matter on glucose homeostasis and target organ damage in a type 1 diabetic rat model. Toxicol. Appl. Pharmacol..

[42] Veras M.M., Damaceno-Rodrigues N.R., Guimarães Silva R.M., Scoriza J.N., Saldiva P.H., Caldini E.G., Dolhnikoff M. (2009). Chronic exposure to fine particulate matter emitted by traffic affects reproductive and fetal outcomes in mice. Environ. Res..

[43] Baron P.A., Willeke K. (2001). Aerosol Measurement: Principles, Techniques, and Applications.

[44] Patashnick H., Rupprecht E.G. (1991). Continuous PM-10 Measurements Using the Tapered Element Oscillating Microbalance. J. Air Waste Manag. Assoc..

[45] Percie du Sert N., Hurst V., Ahluwalia A., Alam S., Avey M.T., Baker M., Browne W.J., Clark A., Cuthill I.C., Dirnagl U. (2020). The ARRIVE guidelines 2.0: Updated guidelines for reporting animal research. PLoS Biol..

[46] Festing M.F.W. (2014). The extended statistical analysis of toxicity tests using standardised effect sizes (SESs): A comparison of nine published papers. PLoS ONE.

[47] Lazic S.E., Clarke-Williams C.J., Munafò M.R. (2018). What exactly is ‘N’ in cell culture and animal experiments?. PLoS Biol..

[48] Warheit D.B., Boatman R., Brown S.C. (2015). Developmental toxicity studies with 6 forms of titanium dioxide test materials (3 pigment-different grade & 3 nanoscale) demonstrate an absence of effects in orally-exposed rats. Regul. Toxicol. Pharmacol..

[49] American Veterinary Medical Association (2020). AVMA Guidelines for the Euthanasia of Animals: 2020 Edition.

[50] Zin S.R.M., Alshawsh M.A., Mohamed Z. (2022). Multiple Skeletal Anomalies of Sprague Dawley Rats following Prenatal Exposure to *Anastatica hierochuntica* as Delineated by a Modified Double-Staining Method. Children.

[51] Wise L.D., Winkelmann C.T. (2009). Micro-computed tomography and alizarin red evaluations of boric acid-induced fetal skeletal changes in Sprague-Dawley rats. Birth Defects Res. B Dev. Reprod. Toxicol..

[52] Barbe M.F., Loomis R., Lepkowsky A.M., Defreitas T., Iqbal S.A., Pechey R., Renner K. (2014). Micro-computed tomography assessment of vertebral column defects in retinoic acid-induced rat model. Birth Defects Res. A Clin. Mol. Teratol..

[53] Fedorov A., Beichel R., Kalpathy-Cramer J., Finet J., Fillion-Robin J.C., Pujol S., Bauer C., Jennings D., Fennessy F., Sonka M. (2012). 3D Slicer as an image computing platform for the Quantitative Imaging Network. Magn. Reson. Imaging.

[54] Zhang Y., Feng H., Zhao Y., Zhang S. (2024). Exploring the Application of the AI-Integrated Platform 3D Slicer in Medical Imaging Education. Diagnostics.

[55] Bouxsein M.L., Boyd S.K., Christiansen B.A., Guldberg R.E., Jepsen K.J., Müller R. (2010). Guidelines for assessment of bone microstructure in rodents using micro-computed tomography. J. Bone Miner. Res..

[56] Groten J.P., Schoen E.D., Feron V.J. (1996). Use of factorial designs in combination toxicity studies. Food Chem. Toxicol..

[57] Narotsky M.G., Weller E.A., Chinchilli V.M., Kavlock R.J. (1995). Nonadditive developmental toxicity in mixtures of trichloroethylene, Di(2-ethylhexyl) phthalate, and heptachlor in a 5 x 5 x 5 design. Fundam. Appl. Toxicol..

[58] Hothorn L.A., Kluxen F.M. (2019). Robust multiple comparisons against a control group with application in toxicology. arXiv.

[59] Crowe A.R., Yue W. (2019). Semi-quantitative Determination of Protein Expression using Immunohistochemistry Staining and Analysis: An Integrated Protocol. Bio Protoc..

[60] Langsrud Ø (2003). ANOVA for unbalanced data: Use Type II instead of Type III sums of squares. Stat. Comput..

[61] Lenth R.V. (2024). emmeans: Estimated Marginal Means, aka Least-Squares Means; R package version 1.11.2.

[62] Fox J., Weisberg S. (2019). An R Companion to Applied Regression.

[63] Wickham H. (2016). ggplot2: Elegant Graphics for Data Analysis.

[64] Barker D.J. (2007). The origins of the developmental origins theory. J. Intern. Med..

[65] Gluckman P.D., Hanson M.A., Cooper C., Thornburg K.L. (2008). Effect of in utero and early-life conditions on adult health and disease. N. Engl. J. Med..

[66] Holson R., Pearce B. (1992). Principles and pitfalls in the analysis of prenatal treatment effects in multiparous species. Neurotoxicol. Teratol..

[67] Galecki A., Burzykowski T. (2013). Linear Mixed-Effects Models Using R: A Step-by-Step Approach.

[68] Zuur A.F., Ieno E.N., Walker N.J., Saveliev A.A., Smith G.M. (2009). Mixed Effects Models and Extensions in Ecology with R.

