# Supplementary material for: Maternal Exposure to Wood-Smoke-Derived PM2.5 Is Associated with Delayed Fetal Neurocranial Intramembranous Ossification in a Rat Model
Source: Int J Mol Sci. 2026 Jun 24;27(13):5715. doi: 10.3390/ijms27135715 (PMC13361095; doi:10.3390/ijms27135715)
Supplement: Supplementary file 1 [file ijms-27-05715-s001.zip › Supplementary Materials/Supplementary Document 2.pdf]

SUPPLEMENTARY DOCUMENTS

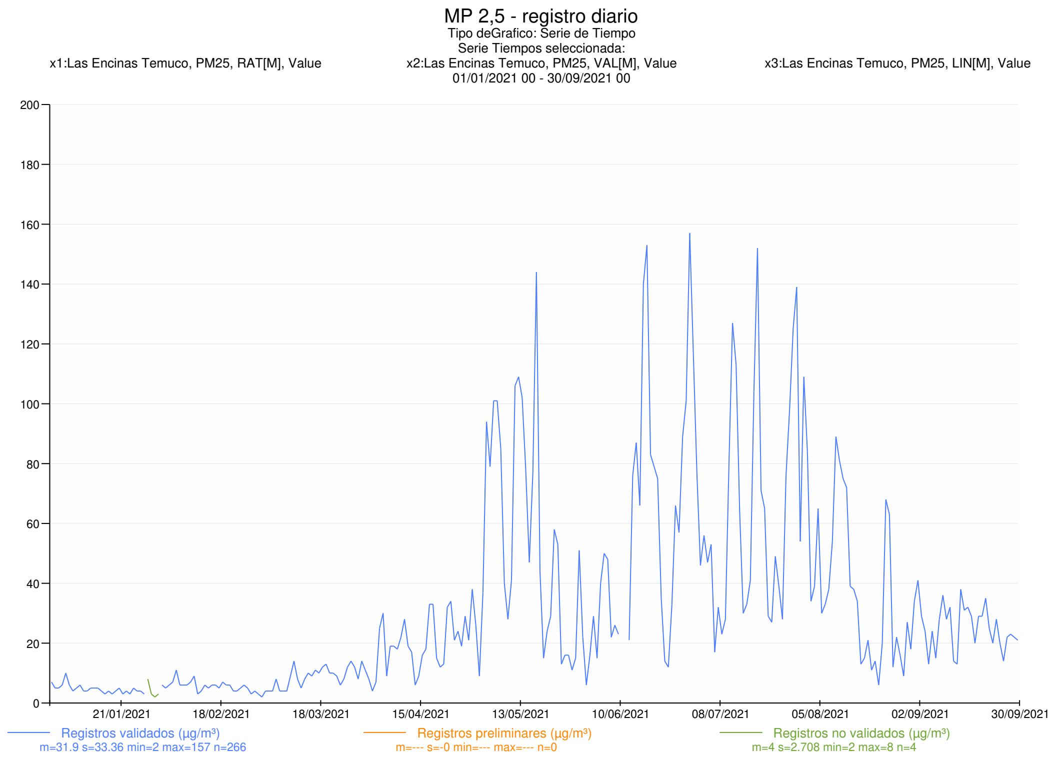


***Supplementary Document 2.*** Figure III. Daily PM2.5 concentrations (µg/m³) recorded at the "Las Encinas Monitoring Station" (Temuco, Chile) during the study period (January 1 to September 30, 2021). PM2.5 levels were measured externally using a beta attenuation monitor BAM 1020 (Met One Instruments, Inc., Grant Pass, OR, USA), equipped with a carbon-14 (60 µCi ±15 µCi) beta source and a photomultiplier tube beta detector with an organic plastic scintillator, operating at a flow rate of 16.7 L/min. Data were provided by "Algoritmos y Mediciones Ambientales SpA" and accessed via the National Air Quality Information System (https://sinca.mma.gob.cl). The chart differentiates between validated (blue), preliminary (orange), and unvalidated (green) records.
